# Correction: Acceptability and feasibility of HIV self-testing among transgender people in Larkana, Pakistan: Results from a pilot project

**DOI:** 10.1371/journal.pone.0316739

**Published:** 2024-12-30

**Authors:** Arshad Altaf, Muhammad Safdar Kamal Pasha, Ayesha Majeed, Wajid Ali, Ahmed Sabry Alaama, Muhammad Shahid Jamil

The copyright statement is missing information. The correct statement is: "© 2022 World Health Organization. Licensee Public Library of Science. This is an open access article distributed under the Creative Commons Attribution IGO License, which permits unrestricted use, distribution, and reproduction in any medium, provided the original work is properly cited. http://creativecommons.org/licenses/by/3.0/igo/. In any use of this article, there should be no suggestion that WHO endorses any specific organisation, products or services. The use of the WHO logo is not permitted. This notice should be preserved along with the article’s original URL."

## References

[1] Altaf A, Pasha MSK, Majeed A, Ali W, Alaama AS, Jamil MS (2022) Acceptability and feasibility of HIV self-testing among transgender people in Larkana, Pakistan: Results from a pilot project. PLOS ONE 17(7): e0270857. doi: 10.1371/journal.pone.0270857 35802646 PMC9269381

