# Supplementary material for: Acceptability and feasibility of HIV self-testing among transgender people in Larkana, Pakistan: Results from a pilot project
Source: PLoS One. 2022 Jul 8;17(7):e0270857. doi: 10.1371/journal.pone.0270857 (PMC9269381; doi:10.1371/journal.pone.0270857)
Supplement: S1 File — (ZIP) [file pone.0270857.s001.zip › Supporting files/3001-3031 0118 OQ Thai ST IFU_PI ENG (for reference).pdf]

ENGLISH

ORAQUICK®
HIV SELF-TEST

VIEW INSTRUCTIONS
www.oraquickhivselftest.com

INSTRUCTIONS FOR USE

You must follow the test directions carefully to get an accurate result. Do not eat or drink for at least 15 minutes before you start the test or use mouth cleaning products 30 minutes before you start the test.

WARNING: If you are on HIV treatment (ARVs) you may get a false result.

HOW TO USE THE ORAQUICK® HIV SELF-TEST KIT

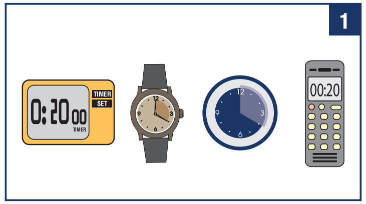

YOU WILL NEED A WAY TO TIME THE TEST

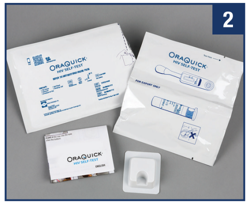

Pouch contains: **test kit, test stand** and **instructions for use.**

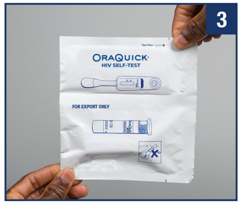

Your test kit contains two pouches.

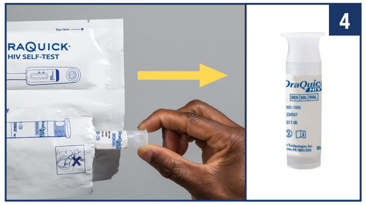

Tear open the pouch containing the **tube.**

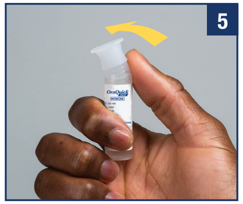

Remove the cap.

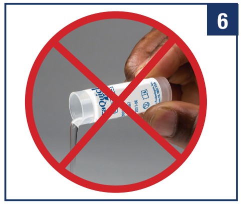

DO NOT pour out the liquid.
DO NOT drink.

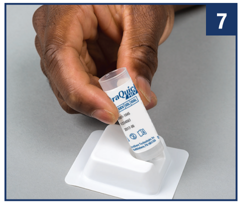

Slide the tube into the **stand.**

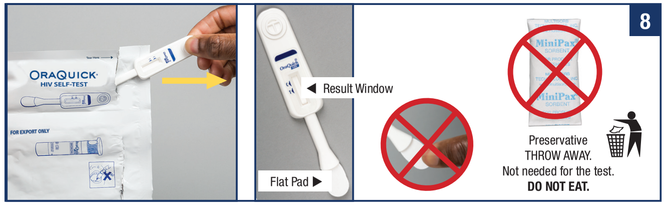

Tear open pouch containing the **test device** and remove. **DO NOT** touch the flat pad with your fingers. **DO NOT** eat or swallow the preservative.

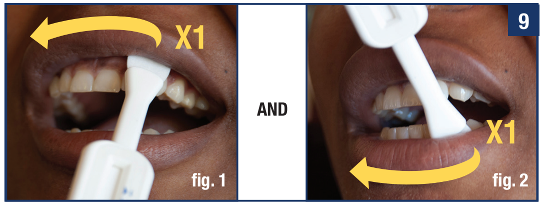

Press the **Flat Pad** firmly against your gum and swab it along your **upper gum once** (fig. 1) and **your lower gum once** (fig. 2).

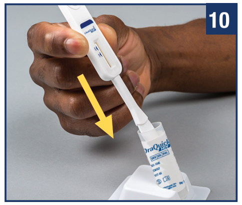

Put the **flat pad** all the way into the tube until it touches the bottom.

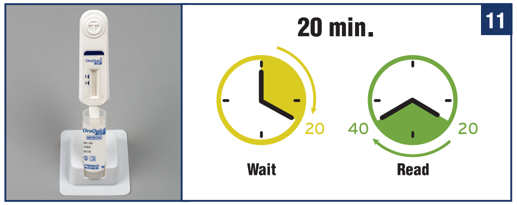

LEAVE IT THERE for 20 MINUTES before reading the results. DO NOT read the result after 40 minutes.

INTERPRETING RESULTS

Read test results in a well-lit area

HIV POSITIVE RESULT

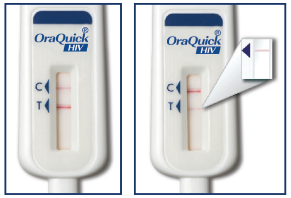

Two complete lines, even if the line is faint, means you may be HIV POSITIVE and you need to seek additional testing.

→ ***As soon as possible . . .***

Visit your nearest HIV Testing Centre or Health Facility

HIV NEGATIVE RESULT

IF READ BEFORE 20 MINUTES, RESULT MAY NOT BE CORRECT

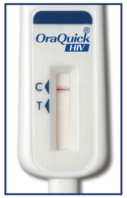

ONE LINE next to the "C" and NO line next to the "T", your result is HIV NEGATIVE.

→ Seek regular testing. If you may have been exposed to HIV, test again in 3 months.

INVALID RESULT

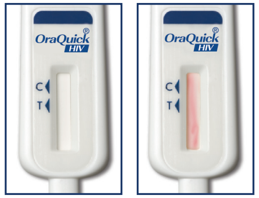

No line next to the "C" (even when there is a line next to the "T"), or a red background makes it impossible to read the test, the test is not working and should be repeated.

You will need to obtain another test.

→ The test did not work properly.

Visit your nearest HIV Testing Centre or Health Facility to test again.

NOT SURE OF RESULT

You do not know your result or you are unsure of your result.

→ Visit your nearest HIV Testing Centre or Health Facility to test again.

DISPOSE

Remove the test stick, put the cap on the test tube and throw away all contents in the normal trash.

Item# 3001-3031-70
rev. 01/18

PRODUCT INFORMATION

REF 5X4-1000, 5X4-1001, 5X4-2001

For Outside USA Use Only
***In Vitro* Diagnostic Use • Do Not Reuse**

INTENDED USE

The OraQuick® HIV Self-Test is an *in-vitro* diagnostic medical device (IVD) that is used for self-testing of antibodies for HIV-1 and HIV-2 in oral fluid. This test is intended as an aid to detect antibodies to HIV-1 and HIV-2 from infected individuals.

SUMMARY OF THE TEST

The OraQuick® HIV Self-Test is a single-use, qualitative immunoassay to detect antibodies to Human Immunodeficiency Virus Type 1 (HIV-1) and Type 2 (HIV-2) in oral fluid. The OraQuick® HIV Self-Test is intended for use by lay users as a self-test to aid in the diagnosis of infection with HIV-1 and HIV-2. The device is placed into the mouth, so that the flat pad is between the cheek and the outer gums, then swabbed across the outer gum line. The device is then placed into a tube containing a premeasured amount of solution. Fluid from the surface of the gums enters the device through the flat pad, then flows onto a test strip. As it flows across the strip, a colored line forms in the 'T' (test) area of the result window if HIV antibodies are detected. If no HIV antibodies are detected, no line forms there. If the test is performed correctly, a line forms in the 'C' area of the result window.
This is called the control line.

TEST PERFORMANCE

In a clinical study, 900 people who were unaware of their HIV status were given the OraQuick® HIV Self-Test to use. The results were compared to a 4th generation laboratory test. The laboratory results show that a total of 153 people were HIV positive and 724 people were HIV negative. Seven (7) people were excluded from the study. The comparison of results was as follows:

- 99.4% of people (152 out of 153) correctly reported their result as positive. This means that 1 out of 153 people infected with HIV reported a negative test result. This is called a false negative.
- 99.0% of people (717/724) correctly reported their result as negative. This means that 7 out of 724 people not infected with HIV reported a positive test result. This is called a false positive.
- In addition, only 1.8% of study subjects (16 out of 900) failed to obtain a test result.

KIT CONTENTS

- One pouched kit containing:
  - Divided Pouch (5X4-0004) with single use Test Device, Preservative and a Developer Solution Vial
  - Test Stand
  - Instructions for Use

Materials required but not provided: Clock, watch, or timing device

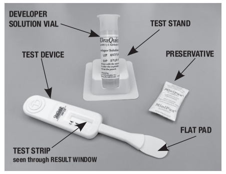

⚠ WARNINGS AND PRECAUTIONS

- Most people feel a little bit nervous when taking an HIV test. But, if you feel very nervous about taking the test, you may want to wait until you are calmer to take it, or get tested by your doctor or local clinic.
- **DO NOT** use the test if you are HIV positive.
- Use with oral fluid only. The test is not for use with blood, serum, breast milk, plasma, semen, urine, vaginal fluid or sweat.
- **DO NOT** eat or drink for at least 15 minutes before starting the test.
- **DO NOT** use mouth cleaning products (such as mouthwash) 30 minutes before starting the test.
- Remove dental products such as dentures or any other products that cover your gums prior to the oral fluid collection.
- If the tamper-evident seal is broken or if any of the package contents are missing, broken, or open, do not use this test.
- If today is after the 'Use By' on the outside of the pouch, do not use this test.
- Individuals must have adequate lighting to read a test result. If two lines are present at areas marked "T" and "C" on the Test Device at any visible intensity, the test result is interpreted as positive.
- **DO NOT** open any of the pouches until you are ready to begin your test.
- **DO NOT** use the test if it has been exposed to household cleaning products (i.e. bleach).
- If you have participated in a HIV vaccine clinical trial, you may get a positive result using this test, but it may not mean that you are infected with HIV. You should seek follow-up with your health facility.
- **DO NOT** use this test if you are age 11 or younger.

30 °C 2 °C **STORAGE**

- Store and perform this test in a cool area.
- **DO NOT** use this test if it has been stored outside the acceptable temperature of 2°-30° C (36°-86° F).
- This test should be performed at temperatures in the range of 15°-37° C (59°-99° F).

LIMITATIONS OF THE TEST

- The OraQuick® HIV Self-Test kit Instructions for use must be followed carefully to get an accurate result.
- If you are on HIV treatment (ARVs) you may get a false result.
- If you are HBV, HCV or HTLV (I/II) positive, you may get a false result.
- Oral bleeding may result in an invalid result. If the test result is invalid, visit your nearest testing center or healthcare facility.
- Clinical data has not been collected to demonstrate the performance of OraQuick® HIV Self-Test in individuals that are undergoing PrEP.
- The OraQuick® HIV Self-Test may not detect HIV infections that have occurred within the last 3 months.
- For a positive result, the intensity of the test line does not necessarily equal the amount of antibody in the specimen.
- Positive results should be verified using another test performed by a trained professional to confirm an HIV diagnosis.

QUESTIONS & ANSWERS

1. ***What does the test do?***
   The OraQuick® HIV Self-Test is an in-vitro diagnostic self-test for HIV (HIV-1 and HIV-2) in oral fluid. The test works by detecting your body's natural antibodies that help you fight infection. A positive result is preliminary and additional testing at a health facility is required to confirm the result as true.
2. ***What is a 'risk event ' for HIV?***
   A risk event is defined by any of the below activities:
   - Sex (vaginal, oral or anal) with multiple sex partners
   - Sex with someone who is HIV positive or whose HIV status you don't know
   - Sex between a man and another man
   - Using illegal injected drugs or steroids
   - Shared needles or syringes
   - Exchanged sex for money
   - Having been diagnosed or treated for hepatitis, tuberculosis or a sexually transmitted disease like syphilis
3. ***How soon after a risk event can I test myself?***
   You can test any time; if you are using this test earlier than 3 months since a risk event and your test is negative, your result may not be accurate. You should test again 3 months after the risk event to be sure. You can also be tested at a health facility.
4. ***Why shouldn't I use this test right after a risk event?***
   When you have been infected with the HIV virus, your body tries to fight the HIV virus by producing natural antibodies. These antibodies can be found in your oral fluid. It takes your body up to 3 months to make these antibodies at levels that can be detected by this test.
5. ***How accurate is the test?***
   In a clinical study, 900 people who were unaware of their HIV status were given the OraQuick® HIV Self-Test to use. The results were compared to a 4th generation laboratory test. The laboratory results show that a total of 153 people were HIV positive and 724 people were HIV negative. Seven (7) people were excluded from the study. The comparison of results was as follows:
   - 99.4% of people (152 out of 153) correctly reported their result as positive. This means that 1 out of 153 people infected with HIV reported a negative test result. This is called a false negative.
   - 99.0% of people (717/724) correctly reported their result as negative. This means that 7 out of 724 people not infected with HIV reported a positive test result. This is called a false positive.
   - In addition, only 1.8% of study subjects (16 out of 900) failed to obtain a test result.
6. ***Can I get HIV by using this test?***
   This test does not contain any materials or HIV virus that can cause HIV infection.
7. ***How often should someone test for HIV?***
   If you have never been tested for HIV, you should be tested at least once. If you do things (risk events) that can result in HIV infection you should be tested at least once per year (World Health Organization recommendation).
   If you feel you are at increased risk for being infected with HIV, you should test regularly.
8. ***What does a negative result mean?***
   A negative result means that the test has not detected any antibodies; however, it may take up to 3 months from a risk event for the test to detect HIV. If it has been **at least 3 months** since you had a risk event and you followed the instructions for use carefully, you likely do not have HIV. If it has been less than **3 months** since you had a risk event, **wait the full 3 months** since the risk event to take the test or go to your health facility.
9. ***What should I do if I get a negative result?***
   If you have not had any risk events within the past **3 months**, and you followed the Instructions for use carefully, then you are most likely HIV negative. If you did not follow the Instructions for use carefully, you should take the test again to be sure your result is correct. If you had any risk events in the past **3 months**, you could be in the 'window period'. The window period is when a person has been infected with HIV, but their body has not made antibodies yet. **If you think you may have been exposed to HIV within the past 3 months**, you should retest for HIV **3 months** following any risk event. If you continue to engage in risk events that could put you at risk for HIV, you should test on a regular basis.
10. ***What does a positive result mean?***
    A positive result means that you may have HIV. **Additional testing must be conducted at a health facility to confirm the result.**
11. ***What should I do if I get a positive result?***
    You need to follow up with a health facility to get additional testing to confirm the result. At that time your local clinic, doctor, or healthcare professional will discuss the next steps that need to be taken.
12. ***Can I get an incorrect 'false' negative result with this test?***
    An incorrect 'false' negative result can occur for any of the following reasons:
    - If you had a risk event less than 3 months prior to taking the test
    - Incorrectly reading test result as negative
    - Not following the instructions for use carefully
    - If you wore dental products such as dentures or any other products that cover your gums while swabbing your gums
    - If you are taking an oral PrEP regimen or if you are on HIV treatment (ARV)
13. ***Can I get an incorrect 'false' positive result with this test?***
    An incorrect or 'false' positive result can occur for any of the following reasons:
    - Incorrectly reading test result as positive
    - Not following the instructions for use carefully
    - Not waiting 15 minutes after eating, drinking, or 30 minutes after using oral care products before taking the test
    - Having received an HIV vaccine
    - Swiping each gum several times during oral collection
14. ***Where can I get additional help or care for HIV?***
    You can get additional help through a local clinic, doctor, or healthcare professional.
15. ***Can I use this test if I am taking medicine to prevent HIV (oral PrEP)?***
    If you are taking oral PrEP for HIV, you may get false result.
16. ***How can I tell if my test is working correctly?***
    If your test is working correctly you will see a line next to the "C" on your test device. If there is no line next to the "C" your test did not work.
17. ***Can I use this test if I am pregnant?***
    Yes, if you are pregnant you can test at anytime.

INTERFERING SUBSTANCES AND UNRELATED MEDICAL CONDITIONS

As part of the oral fluid clinical studies, information was collected from the participants regarding concurrent diseases or medical conditions, oral pathologies, non-HIV viral infections, and other factors (e.g., use of tobacco products, mouthwash within 24 hours of testing, concomitant medications, dental fixtures, and food or drink immediately prior to testing). In a separate study of 40 individuals, consumption of alcohol, brushing of teeth, use of mouthwash or smoking tobacco 5 minutes prior to testing, were shown to have no effect on test specificity. **If you are HBV, HCV or HTLV (I/II) positive, you may get a false result.** It is recommended that users observe a 15 minute wait period after food and drink and a 30 minute wait period after using oral care products.

EXPLANATION OF SYMBOLS

LOT	Batch Code	REF	Catalog Number	⚠	Caution, Consult Accompanying Documents		Consult Instructions for Use
	Do Not Reuse	IVD	*In Vitro* Diagnostic Medical Device		Manufacturer	EXP	Date of Expiration
	Temperature Limitation		Use By			DOM	Date of Manufacturing

Manufactured in Thailand for

OraSure Technologies, Inc.
220 East First Street,
Bethlehem, PA 18015 USA
(+1) 610-882-1820
www.OraSure.com

© 2017, 2018 OraSure Technologies, Inc. • OraQuick®, logo design, and configuration are trademarks of OraSure Technologies, Inc.

Item# 3001-3031 rev. 01/18
