# Supplementary material for: Acceptability and feasibility of HIV self-testing among transgender people in Larkana, Pakistan: Results from a pilot project
Source: PLoS One. 2022 Jul 8;17(7):e0270857. doi: 10.1371/journal.pone.0270857 (PMC9269381; doi:10.1371/journal.pone.0270857)
Supplement: S1 File — (ZIP) [file pone.0270857.s001.zip › Supporting files/Behavioural questionnaire KPs_FINAL.pdf]

سوالنامہ برائے کی پاپولیشن

Demographic and Behavioural Questionnaire for Key Population

اسٹڈی کا نام: ایچ آئی وی سیلف ٹیسٹنگ کٹ تقسیم/ بانٹنے کا پروجیکٹ

Demostration project to determine acceptable distribution model

for HIV self-testing kits among key population in Pakistan

تاریخ:	اسٹڈی نمبر:
شہر:	انٹرویو لینے والے کا نام:
جواب دینے والے کا نام:	موبائل نمبر:

<u>سیکشن نمبر۱: ڈیموگرافی</u>

سوال نمبر۱: عمر<br>(سالوں میں مثلاً 20 یا 21، 21.5 یا 21.4 مت لکھیں)	جواب:
سوال نمبر۲: مادری زبان	۱۔ اُردو<br>۲۔ سندھی<br>۳۔ پنجابی<br>۴۔ بلوچی<br>۵۔ پشتو<br>۶۔ سرائیکی<br>۷۔ کوئی اور (تفصیل لکھیں):
سوال نمبر۳: کتنے سال اسکول گئے ۔	۱۔ اسکول کبھی نہیں گیا/گئی<br>۲۔ سال لکھیں:

PDF created with pdfFactory Pro trial version www.pdffactory.com

سیکشن نمبر۲: رویوں کی تفصیل

سوال نمبر۴: پچھلے تین مہینوں میں آپ کے کتنے جنسی پارٹنر تھے (نمبر لکھیں)	جواب:
سوال نمبر۵: پچھلے تین مہینوں میں آپ کے کس قسم کے جنسی پارٹنرز رہے۔	ا۔مرد<br>۲۔ہیجڑہ<br>۳۔عورت<br>۴۔تینوں
سوال نمبر۶: پچھلے تین مہینوں میں آپ نے پیچھے سے کتنی مرتبہ سیکس کروایا؟ اندازاً نمبر لکھیں۔	جواب:
سوال نمبر۷: پچھلے تین مہینوں میں آپ نے کتنی مرتبہ سیکس کیا جس میں آپ اوپر تھے؟ اندازاً نمبر لکھیں۔	جواب:
سوال نمبر۸: پچھلے تین مہینوں میں آپ نے کتنی عورتوں کے ساتھ سیکس کیا؟ اندازاً نمبر لکھیں۔	ا۔سیکس نہیں کیا۔<br>۲۔نمبر لکھیں:
سوال نمبر۹: آخری دفعہ سیکس کرتے ہوئے کیا آپ نے کنڈوم استعمال کیا؟	ا۔ہاں<br>۲۔نہیں<br>۳۔یاد نہیں
سوال نمبر۱۰: آخری دفعہ سیکس کرتے ہوئے کیا آپ نے لبریکینٹ استعمال کیا؟	ا۔ہاں<br>۲۔نہیں<br>۳۔یاد نہیں

PDF created with pdfFactory Pro trial version www.pdffactory.com

سیکشن نمبر۳: ایچ آئی وی ٹیسٹنگ

سوال نمبر۱۱: آپ نے آخری دفعہ اپنا ایچ آئی وی کا ٹیسٹ کب کروایا تھا؟	۱۔ٹیسٹ نہیں کروایا<br>۲۔ٹیسٹ کروائے ہوئے تین مہینوں سے کم عرصہ ہوا ہے۔<br>۳۔ٹیسٹ کروائے ہوئے تین مہینوں سے زیادہ کا عرصہ ہو گیا ہے۔
سوال نمبر۱۲: آپ نے اپنا ایچ آئی وی کا ٹیسٹ کہاں سے کروایا تھا؟	۱۔اے آر وی سینٹر<br>۲۔سی بی او کے آفس میں<br>۳۔کمیونٹی آؤٹ ریچ ورکر نے کمیونٹی میں کیا<br>۴۔پرائیویٹ لیب سے<br>۵۔یاد نہیں
سوال نمبر۱۳: کیا آپ کے خیال میں آپ ایچ آئی وی کے رسک پر ہیں؟	۱۔ہاں<br>۲۔نہیں<br>۳۔مجھے معلوم نہیں:

PDF created with pdfFactory Pro trial version www.pdffactory.com
