# Supplementary material for: Acceptability and feasibility of HIV self-testing among transgender people in Larkana, Pakistan: Results from a pilot project
Source: PLoS One. 2022 Jul 8;17(7):e0270857. doi: 10.1371/journal.pone.0270857 (PMC9269381; doi:10.1371/journal.pone.0270857)
Supplement: S1 File — (ZIP) [file pone.0270857.s001.zip › Supporting files/Consent_FINAL.pdf]

اجازت نامہ

Consent

اسٹڈی کا نام: ایچ آئی وی سیلف ٹیسٹنگ کٹ تقسیم/ بانٹنے کا پروجیکٹ

Demostration project to determine acceptable distribution model

for HIV self-testing kits among key population in Pakistan

میں یہ بات کنفرم کرتا ہوں کہ مجھے اِس پروجیکٹ کی تفصیل انفارمیشن شیٹ کے ذریعے سمجھا دی گئی ہیں۔ مجھے یہ تمام باتیں اچھی طرح سمجھ میں آ گئی ہیں۔

میں یہ بھی کنفرم کرتا ہوں کہ میری عمر 18 سال یا اس سے زیادہ ہے اور میں اِس پروجیکٹ میں شامل ہو سکتا ہوں۔ میں مزید یہ کنفرم کرتا ہوں کہ:

۱۔ میں ایچ آئی وی نیگیٹیو ہوں یا مجھے اپنا ایچ آئی وی کا اسٹیٹس نہیں پتا۔

۲۔ میں MSM یا TG ہوں۔

۳۔ میں کراچی/ لاڑکانہ میں رہتا ہوں۔

میں نے اِس پروجیکٹ کی تمام تفصیل سمجھ لی ہیں اور میری اس پروجیکٹ میں شمولیت میری مرضی سے ہے۔ میں یہ بھی سمجھ چکا/ چکی ہوں کہ میں جب چاہوں اس پروجیکٹ سے دستبردار (نکل) سکتا ہوں۔

اجازت دے دی____________________

اجازت نہیں دی____________________

PDF created with pdfFactory Pro trial version www.pdffactory.com
