# Supplementary material for: Acceptability and feasibility of HIV self-testing among transgender people in Larkana, Pakistan: Results from a pilot project
Source: PLoS One. 2022 Jul 8;17(7):e0270857. doi: 10.1371/journal.pone.0270857 (PMC9269381; doi:10.1371/journal.pone.0270857)
Supplement: S1 File — (ZIP) [file pone.0270857.s001.zip › Supporting files/HIVST Karachi training opening slides.pdf]

HIV Self-Testing Demonstration Project-Pakistan

Dr Arshad Altaf

World Health Organization

Rationale

- The rate of new HIV infections among key population (KP) is rising in Pakistan
- HIV testing rates and linkages to treatment and prevention is one of the lowest in the Asia Pacific region
- Facility and community-based HTS models are not effectively reaching populations in need of testing
- HIV self-testing (HIVST), a WHO recommended HTS approach, is not implemented in the country

World Health Organization

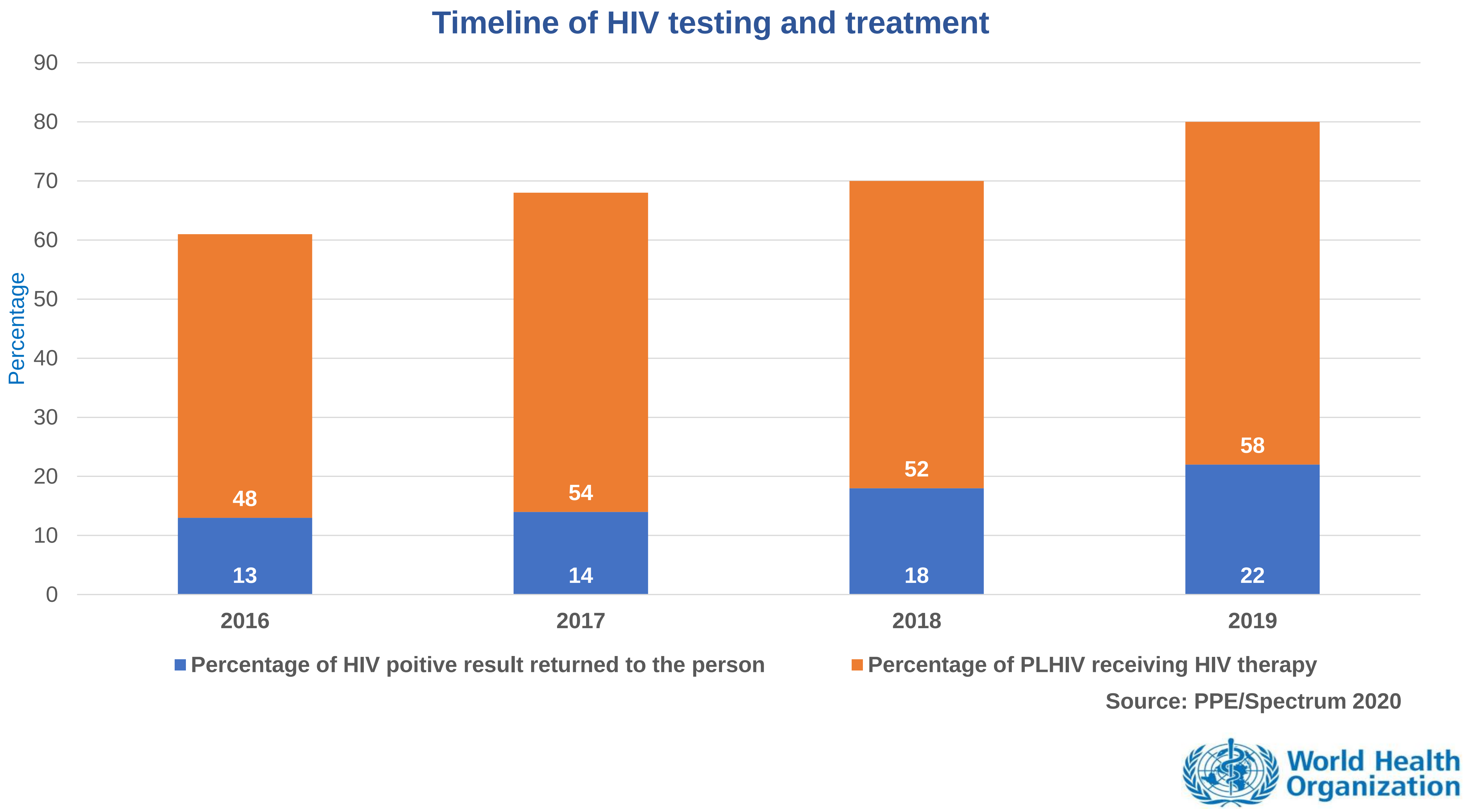
Timeline of HIV testing and treatment
Percentage
90
80
70
60
50
40
30
20
10
0
48
54
52
58
13
14
18
22
2016
2017
2018
2019
Percentage of HIV poitive result returned to the person
Percentage of PLHIV receiving HIV therapy
Source: PPE/Spectrum 2020
World Health Organization

Objectives and outcomes of the demonstration project

Specific objectives

- To pilot HIVST among TG and MSM using two different distribution models including community-based and digital platforms
- To assess feasibility and acceptability of two distribution models among MSM and TGs
- To explore perceptions about self-testing and linkage to care among HIVST users and peer distributors (outreach workers)
- To identify barriers and enablers for HIVST implementation

Outcomes

- Feasibility and acceptability of HIVST distribution models
- Lessons learned for national policy scale up

World Health Organization

Methodology

- Two KP groups: TGs in Larkana and MSM in Karachi
- TGs to be reached using peer outreach approach
- MSM will be approached using digital approaches

Sample size

- No formal sample size calculation conducted; 300 KP persons will be the target
- All those 18 years and above and willing to give consent

Exclusion criteria

- HIV positive or on ART; unable to read instructions; unable or unwilling to provide consent; previous HIVST use

World Health Organization

Progress

- Protocol and tools final (piloted)
- WHO Country office providing support
- Ethical clearance secured
- Testing kits provided by the manufacturer
- Arrival of kits in the country awaited
- Expected within a week
- Trainings will be conducted immediately

انفارمیشن شیٹ۔ اسٹڈی میں شامل حضرات کے لئے

Information Sheet

اسٹڈی کا نام: ایچ آئی وی سیلف ٹیسٹنگ کٹ تقسیم/ بانٹنے کا پروجیکٹ

Demostration project to determine acceptable distribution model for HIV self-testing kits among key population in Pakistan

اجازت نامہ

Consent

اسٹڈی کا نام: ایچ آئی وی سیلف ٹیسٹنگ کٹ تقسیم/ بانٹنے کا پروجیکٹ

Demostration project to determine acceptable distribution model for HIV self-testing kits among key population in Pakistan

سوالنامہ برائے کی پاپولیشن

Demographic and Behavioural Questionnaire for Key Population

اسٹڈی کا نام: ایچ آئی وی سیلف ٹیسٹنگ کٹ تقسیم/ بانٹنے کا پروجیکٹ

Demostration project to determine acceptable distribution model for HIV self-testing kits among key population in Pakistan

تاریخ:	اسٹڈی نمبر:
شہر:	انٹرویو لینے والے کا نام:
جواب دینے والے کا نام:	موبائل نمبر:

World Health Organization

How the findings will help

- HIVST is part of GF country proposal and at the time of grant negotiation in 2021 the demonstration project’s findings will be beneficial for the country’s HIV response
- Findings and lessons learned will help the country in scaling up this approach

What will we do during these two days

- Day 1:
  - Introductions
  - Roles and responsibilities
  - Plenary session covering WHO recommended HIV testing approaches
  - HIV self-testing
  - Using HIV self-testing kits
- Day 2:
  - Recap
  - Walking through all the tools (consent, questionnaire, information sheet and etc)
  - Data issues

World Health Organization

Questions or queries

- altafa@who.int
- mpasha@who.int

World Health Organization
