# Supplementary material for: Acceptability and feasibility of HIV self-testing among transgender people in Larkana, Pakistan: Results from a pilot project
Source: PLoS One. 2022 Jul 8;17(7):e0270857. doi: 10.1371/journal.pone.0270857 (PMC9269381; doi:10.1371/journal.pone.0270857)
Supplement: S1 File — (ZIP) [file pone.0270857.s001.zip › Supporting files/HIVST Karachi training slides.pdf]

Differentiated HIV Testing Approaches for Key Population

Dr Arshad Altaf

World Health Organization

Acronyms

• HTS	HIV testing services
• HIVST	HIV self-testing
• KP	Key population
• LTFU	Lost to follow up
• MSM	Men who have sex with men
• PLHIV	People living with HIV
• SW	Sex worker
• TG	Transgender

Outline

- What is HIV testing service
- Key population (KP)
- Impact of COVID-19 on HIV prevention and testing services
- Challenges faced by KPs in accessing HIV testing services
- Differentiated HIV testing approaches (HTS) for key population

World Health Organization

HIV testing services

Key definition: HIV testing services

The term *HIV testing services* (HTS) is used throughout these guidelines. This term embraces the full range of services that should be provided together with HIV testing. These include brief pre-test information and post-test counselling; linkage to appropriate HIV prevention, care and treatment services and other clinical and support services; and coordination with laboratory services to support quality assurance.

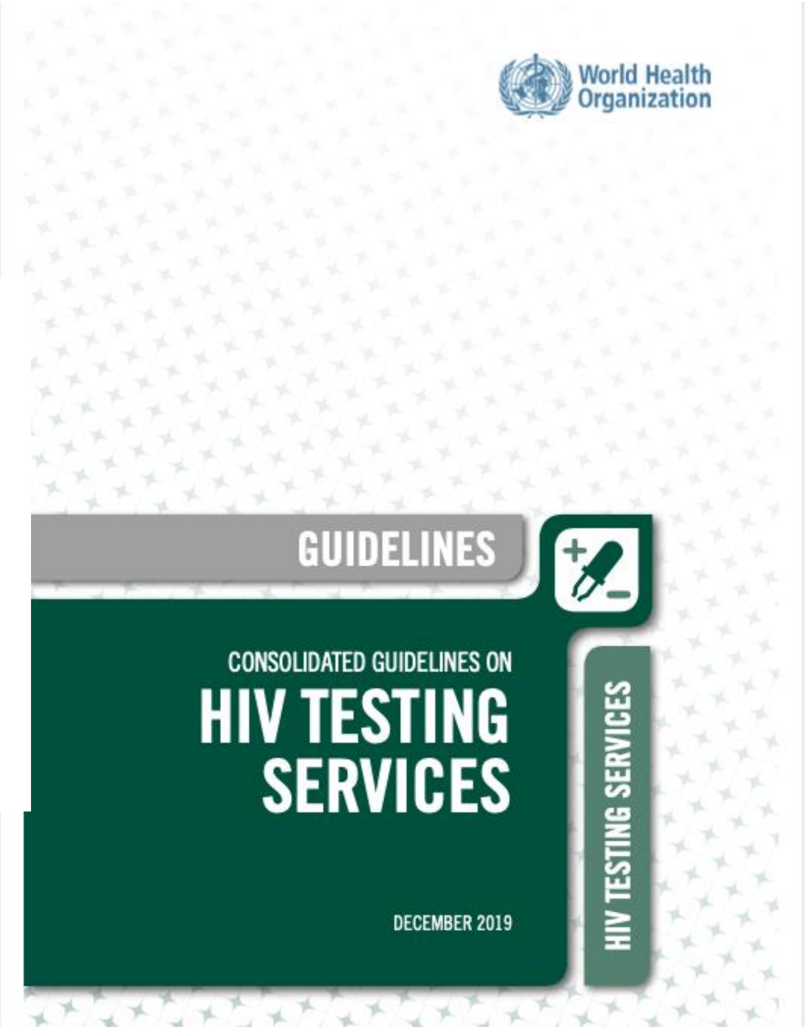

World Health Organization

Key population (KP)

- There is no set definition of key population
- Each country should define the specific populations that are particularly vulnerable and key to their epidemic and response based on the epidemiological and social context
- ***"Key populations are groups of people who are particularly vulnerable to HIV infection in certain situations or contexts"***

Definition of key terms: https://www.who.int/hiv/pub/guidelines/arv2013/intro/keyterms/en/

HIV testing

World Health Organization

Progress towards 90-90-90 targets, global, 2019

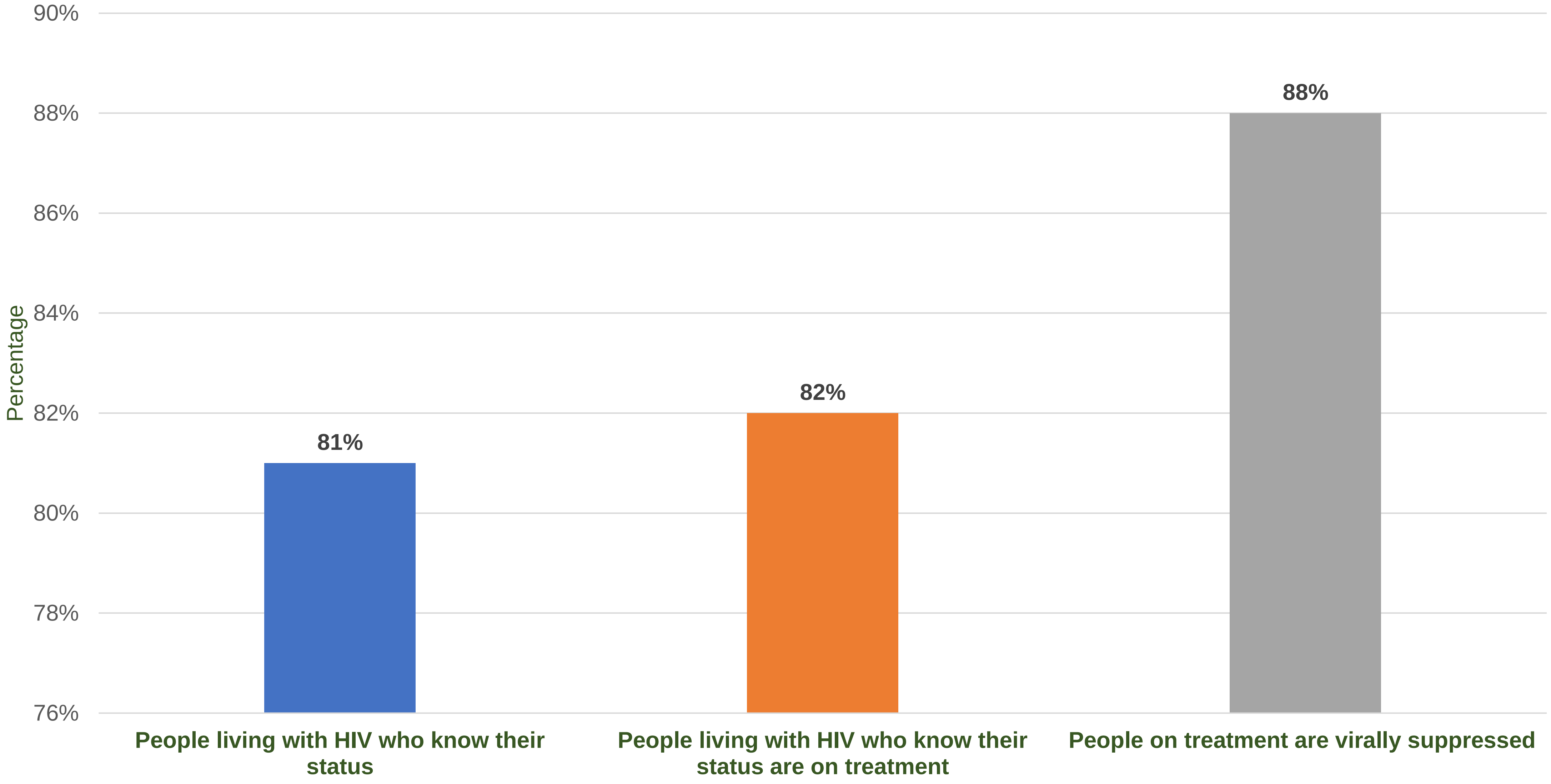

Source: UNAIDS special analysis 2020

World Health Organization

Average HIV testing and status awareness among key populations, global 2016-2019 (Source: UNAIDS GAM 2020)

Significant gaps in HIV testing among key population;

On average about two thirds of KPs Globally had taken an HIV test and received results within the past 12 months

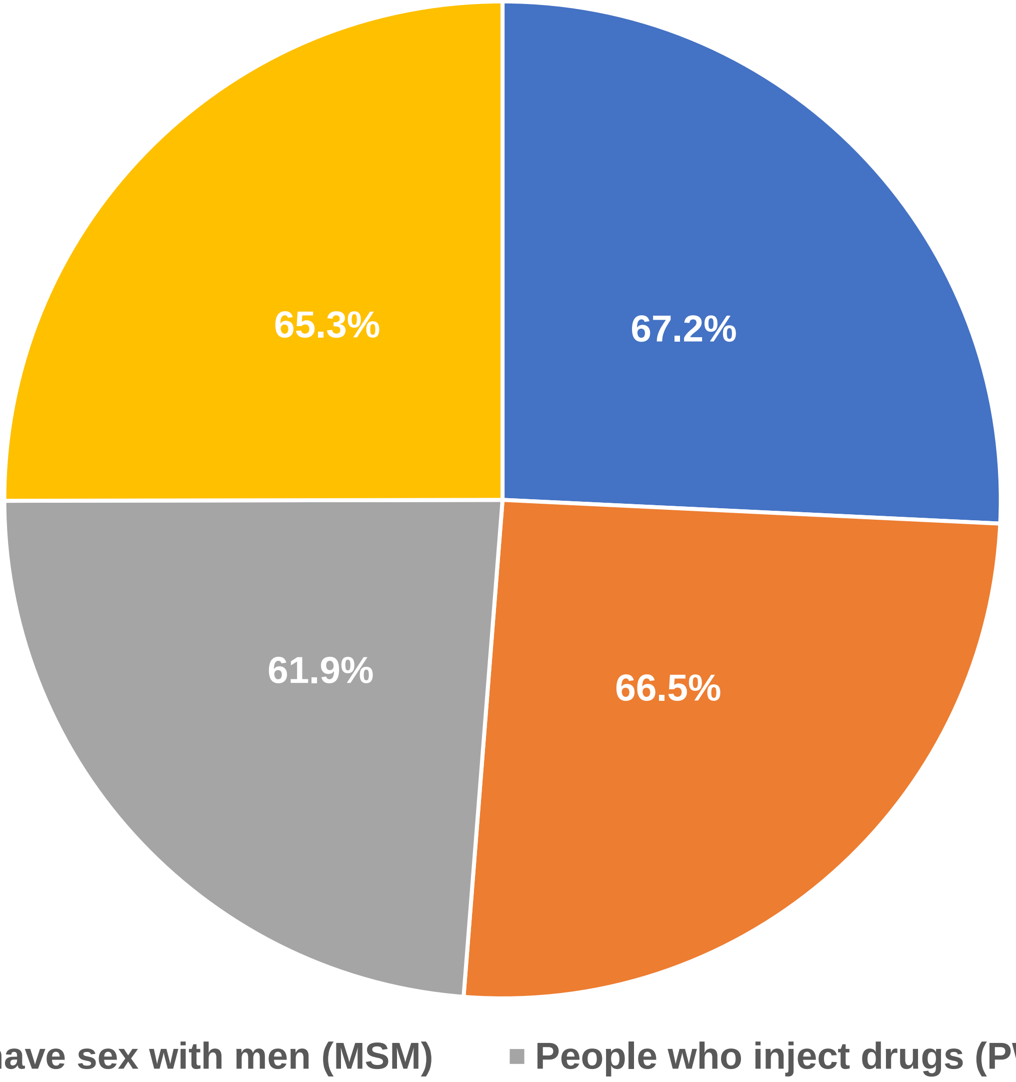

World Health Organization

Key populations account for most HIV infections

Distribution of new HIV infections by gender and population, global, 2019

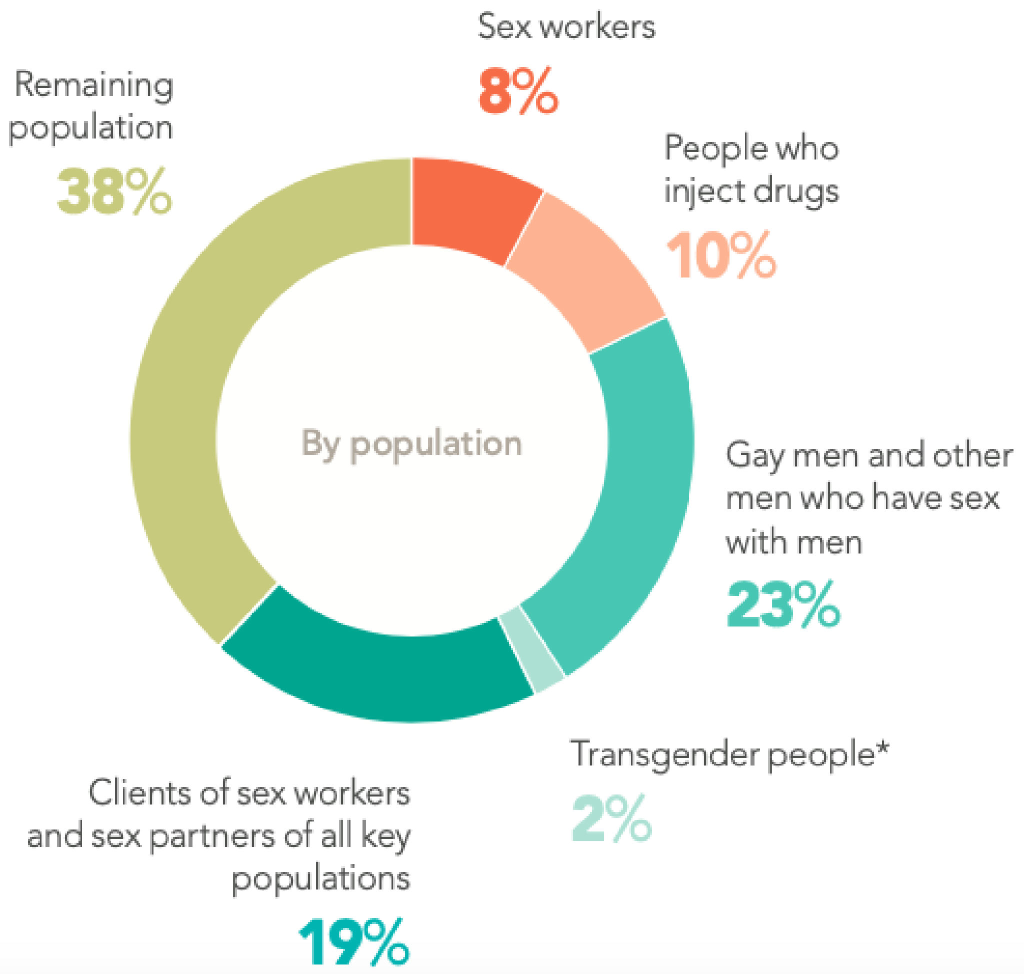

Key population are small portion of the general population but key populations and their partners accounted for 60% of new adult HIV infections globally in 2019

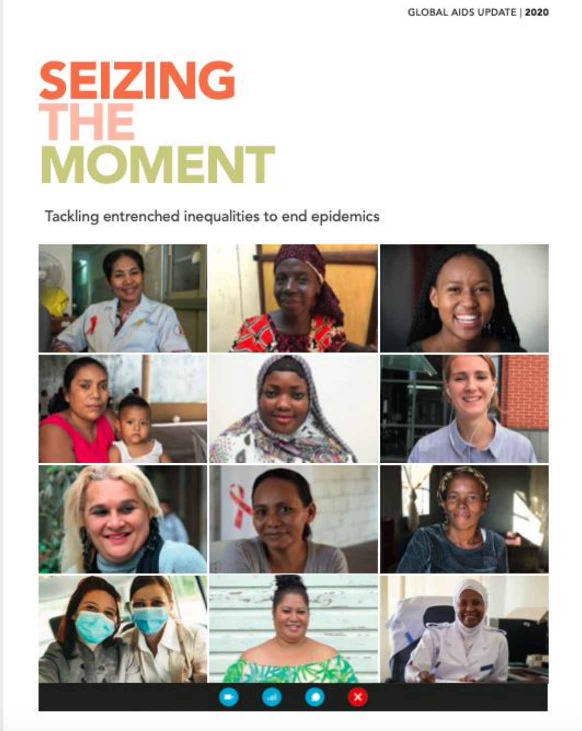

Source: UNAIDS Seizing the moment https://aids2020.unaids.org/report/

World Health Organization

Understanding gap: Who is missing?

- Globally the **21%** of PLHIV undiagnosed are primarily
- Biggest gaps in
  - **Key populations (KP) and their partners (MSM, TG, SW)**
  - **Men** in high HIV burden settings
  - **Young people** (age 15-24) from KP and in high HIV burden settings
  - **FP service** attendees in high HIV burden settings
  - **Partners of PLHIV**
  - **STI patients**
- **LTFU PLHIV** (also need to be tested to be re-engaged in care)

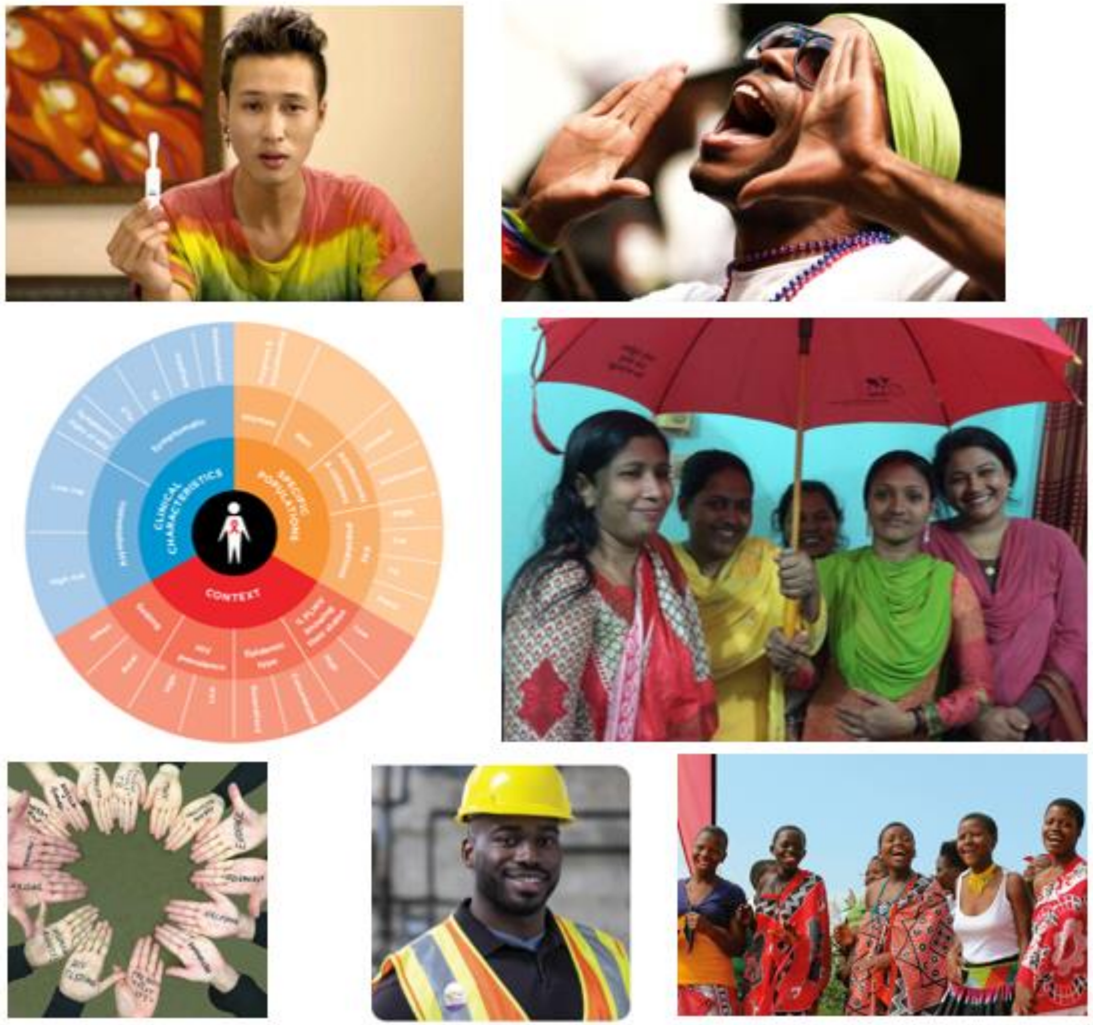

HIV testing and treatment cascade, 2019

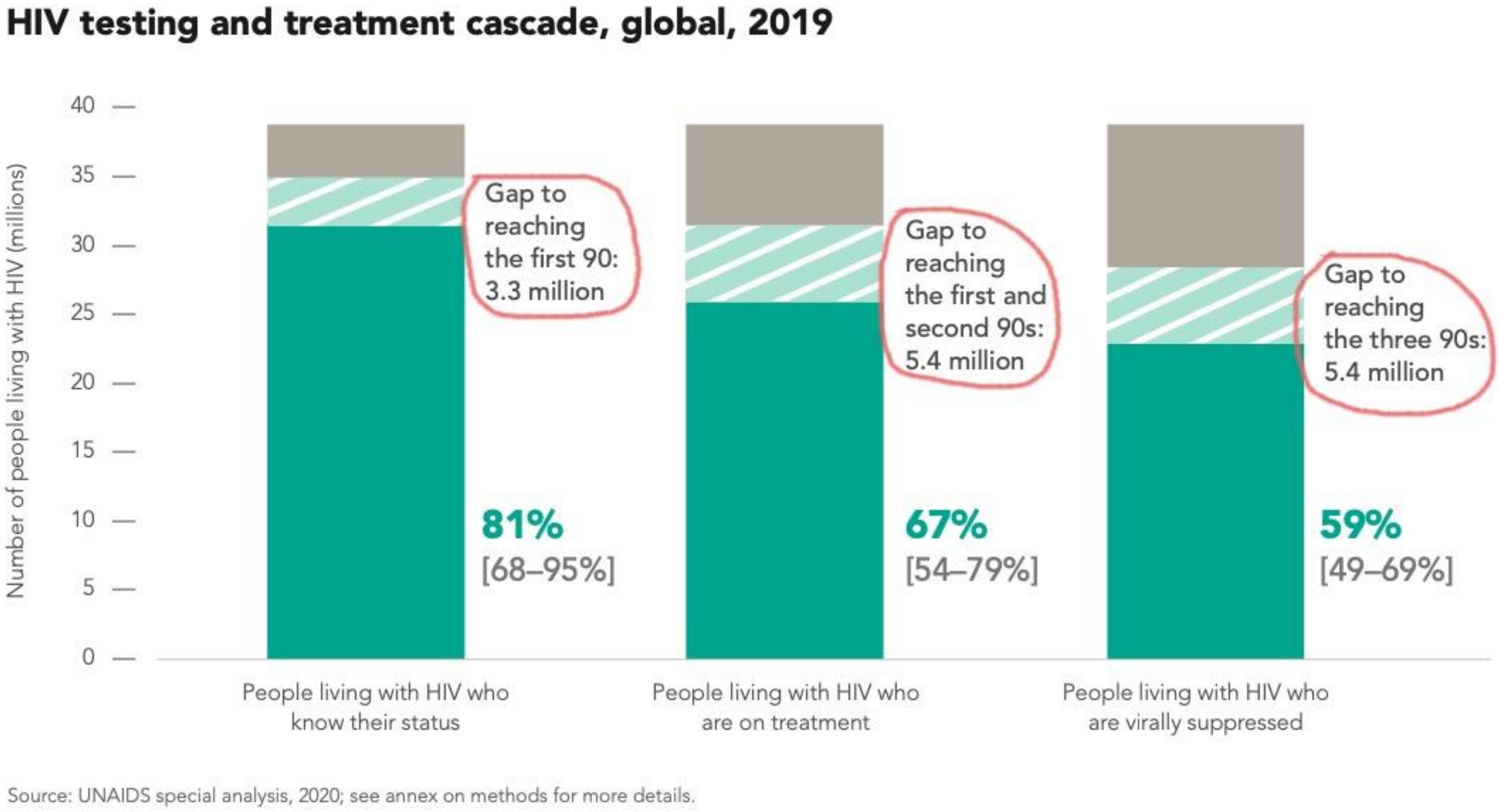

Source: *UNAIDS 2020*

World Health Organization

HIV testing challenges faced by key population

World Health Organization

Key challenges faced by KPs

- Lack of access
- Available and accessible services
- Legal and social issues of key population
- Behavioral issues leading to vulnerability
  - Stigma and discrimination
  - Criminalization
  - Punitive laws
- Low quality of HIV prevention and testing services

References

1) Prevention gap report. Geneva: Joint United Nations Programme on HIV/AIDS; 2016. **2)** Global AIDS update 2019 — Communities at the centre. Geneva: Joint United Nations Programme on HIV/AIDS; 2019. **3)** Brown T, Peerapatanapokin W. Evolving HIV epidemics: the urgent need to refocus on populations with risk. Curr HIV/AIDS Rep. 2019;14(5):337-53. **4)** Are key populations really the 'key' to ending AIDS in Asia? New Dehli: World Health Organization Regional Office for South-East Asia; 2018. **5)** Consolidated guidelines on HIV prevention, diagnosis, treatment and care for key populations. Geneva: World Health Organization; 2016.

Impact of COVID-19 on HIV services

- Global HIV response dependent on generic medicine manufactured in India
  - Lockdown and ease of lockdown restrictions by May 2020 have already resulted in considerable delays in delivery of medicines
- WHO and UNAIDS models predict that six months disruptions in HIV treatment could lead to 300,000 extra AIDS related deaths in sub Saharan Africa over one year period; taking the AIDS related death rates back to 2011 rates
- Pace is off to reach the ”Fast Track Targets” to end the AIDS epidemic
- New investments and revised planning prudent to respond to HIV and COVID-19

Source: COVID 19 and HIV 1) Moment 2) Epidemics 3) Opportunities. UNAIDS 2020.

World Health Organization

COVID-19 affecting key population

- COVID-19 pandemic has affected lives and livelihood everywhere
- Impact is severe among:
  - socioeconomically disadvantaged
  - those with underlying medical condition
  - **key population**
- Sex workers face exploitative working conditions
- COVID-19 lockdowns provide no protection to sex workers causing severe economic difficulties

Source: UNAIDS, Seizing the Moment, Global AIDS Update 2020, page 20

COVID-19 affecting key population contd.

- Lockdown regulations in Panama required movement based on gender i.e. one day only males and another day for females only; resulted in harassment of transgender being treated as a male
- Emergency rules imposed in Hungary restricted people to change their gender identity and names
- Data analysis from eight countries in Africa showed physical distancing difficult due to poverty among PLHIV; living in cramped conditions; share one toilet in their homes

Source: UNAIDS, Seizing the Moment, Global AIDS Update 2020, page 29

World Health Organization

Amid the disruption there is some positive impact of COVID-19

HIVST demonstration project in Manila

- Demonstration project implemented by LoveYourself (NGO) in Metro Manila area
- Clients assisted via a specially developed chat-box for delivery and testing process
- Volunteers provided assistance and counseling
- Condoms and lubricants also included in HIVST kit delivery
- Kits were meant to last six months, but allocated kits were used up within a month of implementation (14 Apr-14 May) due to lockdown
- **4,205** unique clients expressed interest; **2,259** were qualified; **1,689** were successfully delivered; **1,133** reported their results
- **93** were validated reactive (8% reactivity rate); **55** linked to treatment

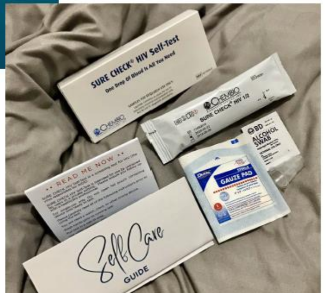

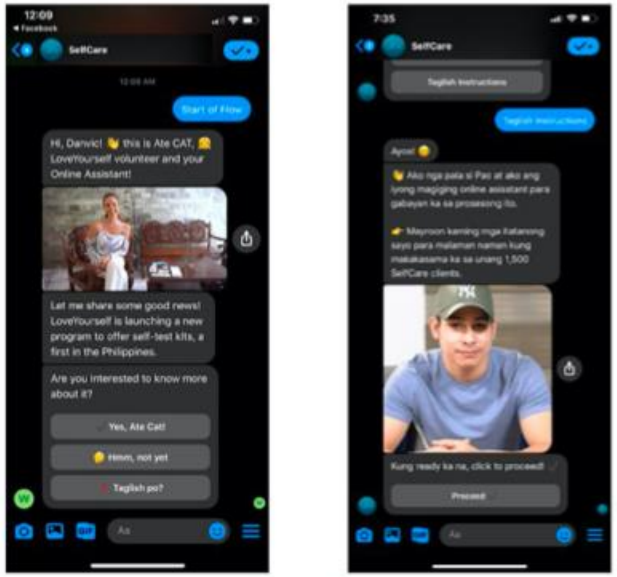

HTS approaches

World Health Organization

HIV tests

There are two main types of test for HIV

- Serologic tests are indirect tests which measure the body's response to an infection. Serologic tests look for antibodies against HIV.
  - All HIV rapid tests are serologic tests.
  - HIV self-testing is also a serologic test.

- Virologic tests are tests which directly measure the presence of HIV in blood.
  - DNA PCR is a qualitative (yes/no) test for HIV.
  - Viral load or RNA PCR is a quantitative (#) test for HIV.

World Health Organization

All forms of HTS should adhere to WHO 5Cs

1. **Consent**: People receiving HTS must give informed consent to be tested and counselled (verbal consent is sufficient).
2. **Confidentiality**: What the HTS provider and the client discuss will not be addressed to anyone without the expressed consent of the person being tested.
3. **Counselling**: Pre-test information and post test counselling can be provided in a group setting if appropriate, all persons should have the opportunity to ask questions in a private setting if they request it.
4. **Correct test results**: HTS providers should make an effort to provide high quality testing service. Quality assurance mechanism should ensure that people receive correct diagnosis. All people who receive a positive diagnosis should be retested before initiation of ART.
5. **Connection**: Linkage to prevention, care and treatment services should include provision of effective follow up including long term prevention and treatment support.

Frequency of HIV testing people (HIV-negatives)

World Health Organization

Primary retesting goals:
1. Enable people who are HIV-negative to **stay HIV-negative** (link or stay engaged in prevention)
2. **Identify new PLHIV** as early as possible so that they can start treatment

Annual retesting, in high HIV burden setting, advised for:
- sexually active individuals in high HIV burden settings and;
- people who have ongoing HIV-related risks in all settings.

More frequent retesting, e.g. every 3–6 months
- based on individual risks and as part of broader HIV prevention interventions, e.g. quarterly testing while taking PrEP, or KP with risks such as an STI
- **(Not recommended to advise retesting every 3-months e.g. "window period for all")**

Retesting in specific groups
- People presenting with a diagnosis or receiving treatment for STIs or viral hepatitis
- People with a confirmed or presumptive TB diagnosis
- outpatients presenting with clinical conditions or symptoms indicative of HIV
- individuals with recent HIV risk exposure or who are concerned that they may have been exposed.

UPDATED GUIDANCE

Accurate HIV diagnosis

WHO standard HIV testing strategy for a changing HIV epidemic

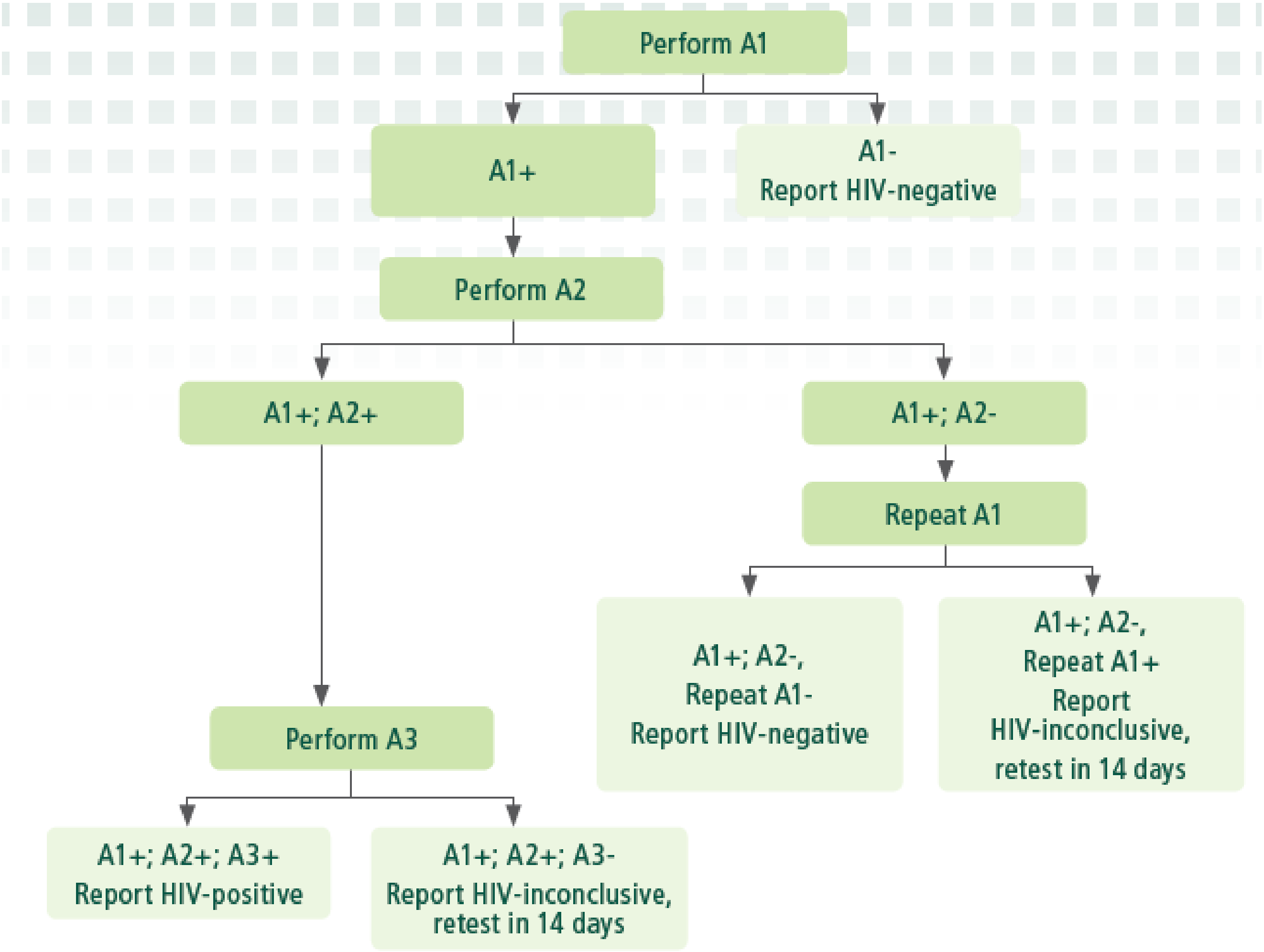

Source: Consolidated guidelines on HIV testing services. Geneva: World Health Organization; 2019.

World Health Organization

New WHO guidelines on HTS for a changing epidemic

8 updates and new recommendations/guidance

Box. 1 Summary of new WHO guidance, recommendations and good practice statements

1. **Demand creation:** NEW Good practice statement highlighting evidence-based approaches and considerations for the use of incentives for HIV testing services, including linkage.
2. **Counselling message:** ✓ Updated messages and guidance on concise communications with emphasis on linkage and latest information on the benefits of treatment and prevention services.
3. **HIV self-testing:** ✓ Updated HIV self-testing should be offered as an approach to HIV testing services *(strong recommendation, moderate-quality evidence)*.
4. **Social network-based approaches:** NEW Social network-based approaches can be offered as an HIV testing approach for key populations as part of a comprehensive package of care and prevention *(conditional recommendation, very low-quality evidence)*.
5. **HIV testing strategies:** ✓ Updated. In response to changes in the HIV epidemic, WHO encourages countries to move toward using three consecutive reactive tests to provide an HIV-positive diagnosis.
6. **Western blotting:** NEW Western blotting and line immunoassays should not be used in national HIV testing strategies and algorithms *(strong recommendation, low-quality evidence)*.
7. **Dual HIV/syphilis rapid diagnostic tests:** All pregnant women should be tested for HIV, syphilis and hepatitis B surface antigen (HBsAg) at least once and as early as possible *(syphilis testing: strong recommendation, moderate-quality evidence; HBsAg: strong recommendation, low-quality evidence)*.

   NEW Dual HIV/syphilis rapid diagnostic tests (RDTs) can be the first test in HIV testing strategies and algorithms in ANC settings.
8. **Optimal maternal retesting time points:** ✓ Updated. In high HIV burden settings, retesting is advised for all pregnant women with an unknown or HIV-negative status during late pregnancy (third trimester). Catch-up testing is needed if the first test or retest is missed or delayed. High HIV burden countries could consider an additional retest in the post-partum period for specific districts or regions with high HIV burden or incidence, women from key populations or who have a partner with HIV who is not virally suppressed.

ld Health
anization

WHO recommended HIV testing service delivery approaches

HTS is an important gateway to treatment and prevention for individuals, partners, couples and families

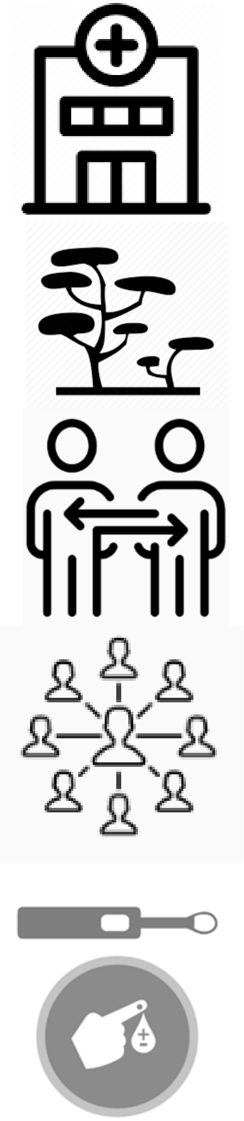

**Facility-based:** Offering HIV testing in a facility, e.g. VCT, in-patient and out-patient clinics, ANC, **TB**, **STI**, **family planning/contraceptive services**
**Community-based**: Offering HIV testing in natural setting of the community, e.g. outreach, CBOs, workplace, clubs, bars.
**UPDATED Provider-assisted referral** (i.e. index testing or assisted partner notification): Assisting individuals with HIV by contacting their sexual and/or drug injecting partners and offering them HIV testing services.
**NEW Social network-based approaches**: whereby key populations offer HTS to their social, sexual and drug injecting partners at risk of HIV. Includes HIV+ and HIV- key populations
**UPDATED HIV self-testing:** Offering self-test kit for individual, and/or their partner, enabling them to collect their sample (oral or blood), perform test, and interpret results in private. All reactive results need confirmation.

MOBILIZING TESTING LINKING

World Health Organization

NEW GOOD PRACTICE STATEMENT

Demand creation for reaching undiagnosed PLHIV

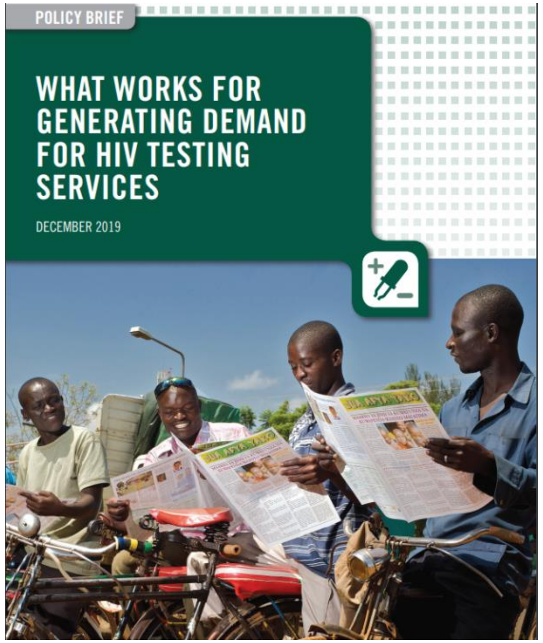

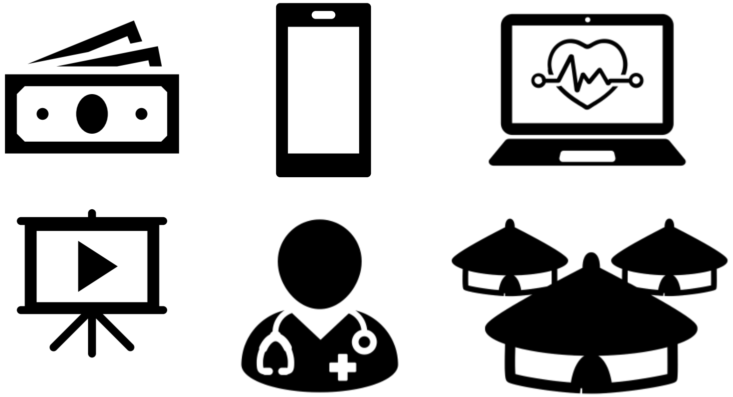

- **Must be focused**. Can be critical for increasing uptake of HTS and linkage to ART and other HIV prevention for those currently being missed.
- 86 RCTs included in the review across diverse populations and settings
  - Interventions included: mobilization, incentives, digital, targeted and tailored and messaging/counselling
- So what works?

World Health Organization

How to deliver HTS demand creation?

Peer-led demand creation ***(moderate quality evidence)***

- **peer-delivered**, participatory and community-led approaches, such as using peer educators, community groups and faith-based programmes **increase HTS uptake**;
- Important to focus on priority populations

Digital platforms and videos ***(moderate quality evidence)***

- **10-fold** increase in HTS uptake with short videos
- Can be considered or adapted based on population and setting
- Costs can be high, but **crowd sourcing and community-engagement** can increase affordability
- May increase efficiency and save health worker time – or be **useful for approaches like HIVST**
- **Social media and web-based tools** limited, but promising esp **young people** and **key populations**

Experience from FHI360 in Viet Nam (March 2016 to January 2019)

- Using online platforms, peer outreach workers counselled **6367 online users**, of **76%**(4879) tested.
- **75% of those contacted had never been in contact with a peer or outreach worker** and 1/3 self-assessed as being at substantial risk for HIV.
- Overall, 431 **(10%) individuals were diagnosed with HIV**. This ***<u>HIV positivity is higher than among key populations</u>*** seeking testing through other referrals (10% versus 6%).

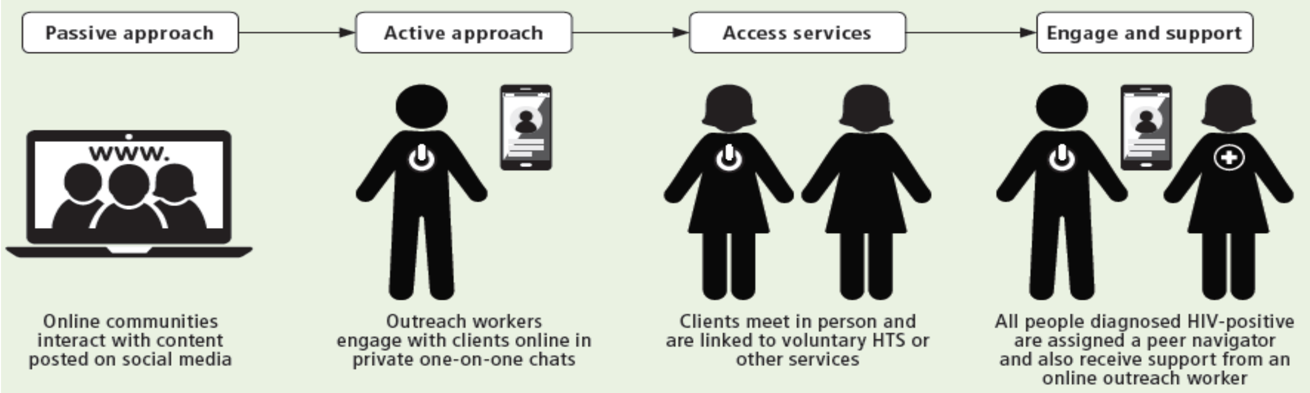

World Health Organization

NEW GOOD PRACTICE STATEMENT

Effective ways to create demand for HTS?

Key approaches supported by evidence:

1. advertisement of specific HTS attributes (especially testing via workplace);
2. brief key messages;
3. messages encouraging testing during couples counselling (including partner services and provider-assisted referral);
4. messages related to risk reduction and economic empowerment, particularly for people who inject drugs;
5. motivational messages.

Approaches that may be less effective:

1. personal invitation letters (alone);
2. individualized content messaging;
3. counselling focused on building relationship between the client and counsellor;
4. general text messages, such as SMS.

Special considerations if thinking about incentives:

- Can improve uptake, but effect on linkage uncertain.
- Issues of sustainability, equity and resource use need to be addressed and its benefits and risks carefully weighed when considering financial incentives for demand creation.

World Health Organization

Facility based HIV testing services

- Facility based HIV testing service means HIV testing in a health facility or laboratory setting
- Facility based HTS can be stand alone often called VCT or at clinical sites often referred to as provider initiated testing and counseling (PITC)

WHO recommendations on routinely offered facility-based HTS

In high HIV burden settings, routine HIV testing should be offered to **all clients** (adults, adolescents and children) in all clinical settings.

In low HIV burden settings, HIV testing should be offered in clinical settings to **clients who present with symptoms or medical conditions** that could indicate HIV infection, including presumed and confirmed TB cases.

In all settings routine HIV testing should be considered for **STI, viral hepatitis, TB, ANC, malnutrition clinics and other health services for key populations.**

Source: WHO, 2007 *(16)*; WHO, 2015 *(1)*.

World Health Organization

Considerations for integrating HTS

- HTS can be integrated into a variety of clinical service; for example:
  - ANC, PNC, contraception/family planning and paediatric services
- Contraception/family planning
  - Provide an opportunity to target adolescent girls and young women of reproductive age and their partners
  - Important in high burden settings
  - Can help in addressing diverse needs of sexually active adolescents
- TB services
  - TB is a leading cause of death among PLHIV
  - Early detection and linkage to TB treatment and ART can prevent deaths among PLHIV
  - All people diagnosed with TB in HIV testing services should be offered TB treatment

Clinical services for key populations

Key population do access health services, when they do, it is important to minimize missed opportunities for HTS

- Drop in centers (DIC)
- Harm reduction services for PWIDs
- Clinical services in prisons

STI clinics provide an important entry point for HTS

- HTS should be offered to all people with STIs

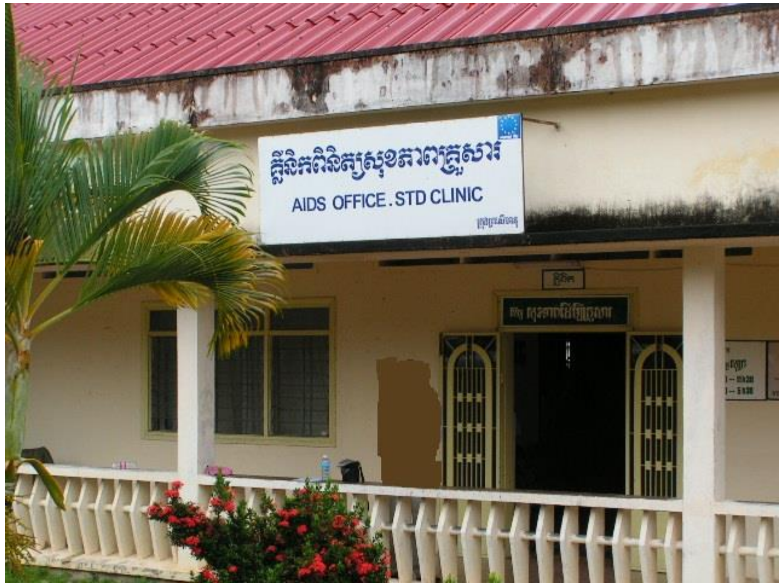

World Health Organization

Social network based (SNA) approach *targeting key population*

- **What is a social network**
  - Group of individuals linked by a common set of relationships or behaviours
  - Can include sexual or drug injecting partners or social contacts of key population
- **What are social network based HIV testing approaches**
  - An extension of HIV partner services
  - Trained provider asks HIV positive person or those at risk of HIV to encourage individuals in their network to participate in HTS
- **Voluntary means**
  - Social network and partner services should be voluntary
  - WHO does not support mandatory or forced partner services or HTS

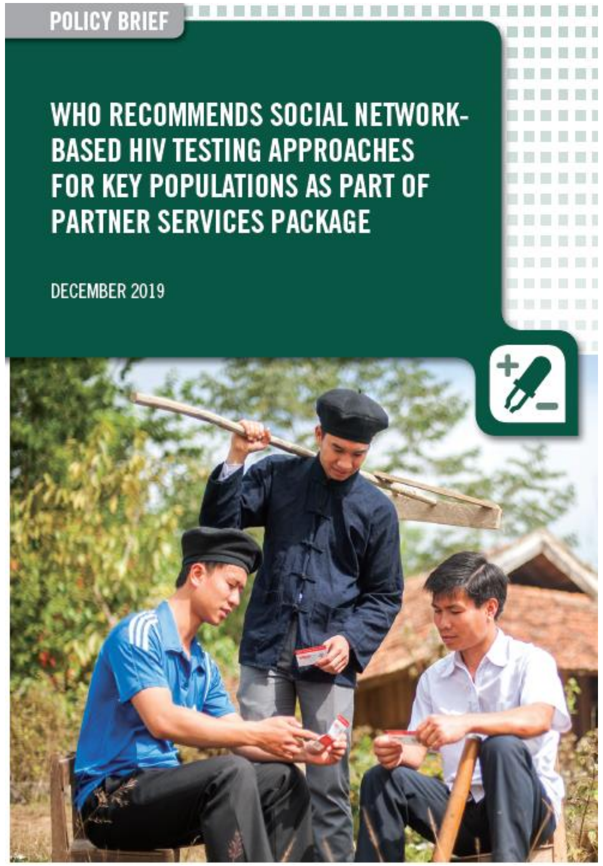

World Health Organization

Key findings from WHO systematic review supporting social network based approach

Evidence from WHO systematic review showed that social network based approaches

- May increase HIV diagnoses and identify additional people with HIV
- May increase the acceptability of HIV partner services
- Are feasible to implement
  - especially among key population particularly HIVST and as part of partner services
- Can be efficient use of resources when focused on key population (FSW, MSM, men, young people)
- Seldom result in social harm or adverse events

World Health Organization

Promising models of SNA
reaching key population

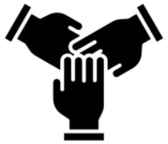

- **Peers**
  - Use of peers to recruit key population in their networks for HTS
- **HIV self-testing kits**
  - Distributing HIVST by HIV positive and HIV negative clients to their partners and contacts

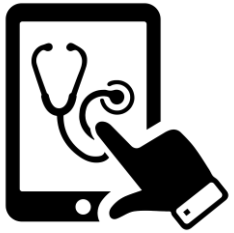

- **Use of technology**
  - Digital and social media to reach social networks especially for young key population
- **Anonymous methods**
  - For partner services and social network based approaches to protect confidentiality of key population experiencing stigma, discrimination and criminalization

World Health Organization

Task sharing/lay providers

- Lay provider is any person who performs functions related to health care delivery and has been trained to deliver a specific set of services
- A lay provider may not have a formal professional or paraprofessional certificate
- Lay providers can work as effective community outreach workers
- Lay providers:
    - can increase uptake of HIV testing
    - can perform task accurately
    - are often preferred
    - will be low cost

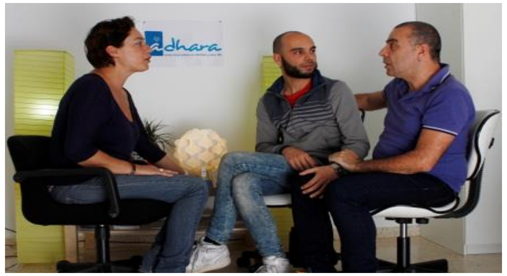

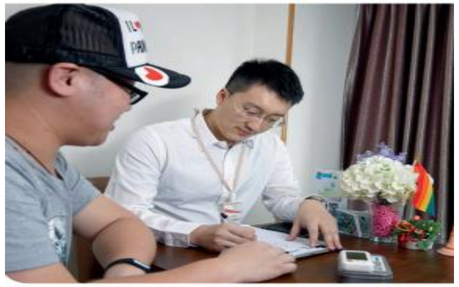

World Health Organization

HIV partner services

- Offering voluntary HIV testing to sexual and/or drug injecting partners of people with HIV
- Options:
  - **Provider assisted referral**: a trained provider directly assists people who have tested HIV positive by contacting their partners
  - **Patient referral**: a trained provider encourages the client to disclose their HIV status to their partners
- Important to offer HIV testing for untested biological children of HIV positive clients

Enhanced patient referral

- Trained provider uses various support tools to facilitate disclosure and offers HTS
- The tools may include:
  - Providing written information
  - Leaflets
  - Referral slip
  - Card for the partner(s)
  - Web based messaging platforms to inform partners anonymously
  - Providing HIVST kits to HIV positive clients to give to their partner(s) to test themselves for HIV

Provider assisted referral

- Trained provider asks people with HIV about their sexual and/or drug injecting partner and
  - with consent of HIV positive client informs the partners of their potential exposure to HIV
  - Offers voluntary HTS
- Provider can contact the partner(s) by telephone, email or in person and offer home based HTS
- Invite them to a facility to receive HTS
- Provider assisted referral provides the opportunity to offer comprehensive prevention interventions to vulnerable partners who are HIV negative

World Health Organization

UPDATED RECOMMENDATION (strong)

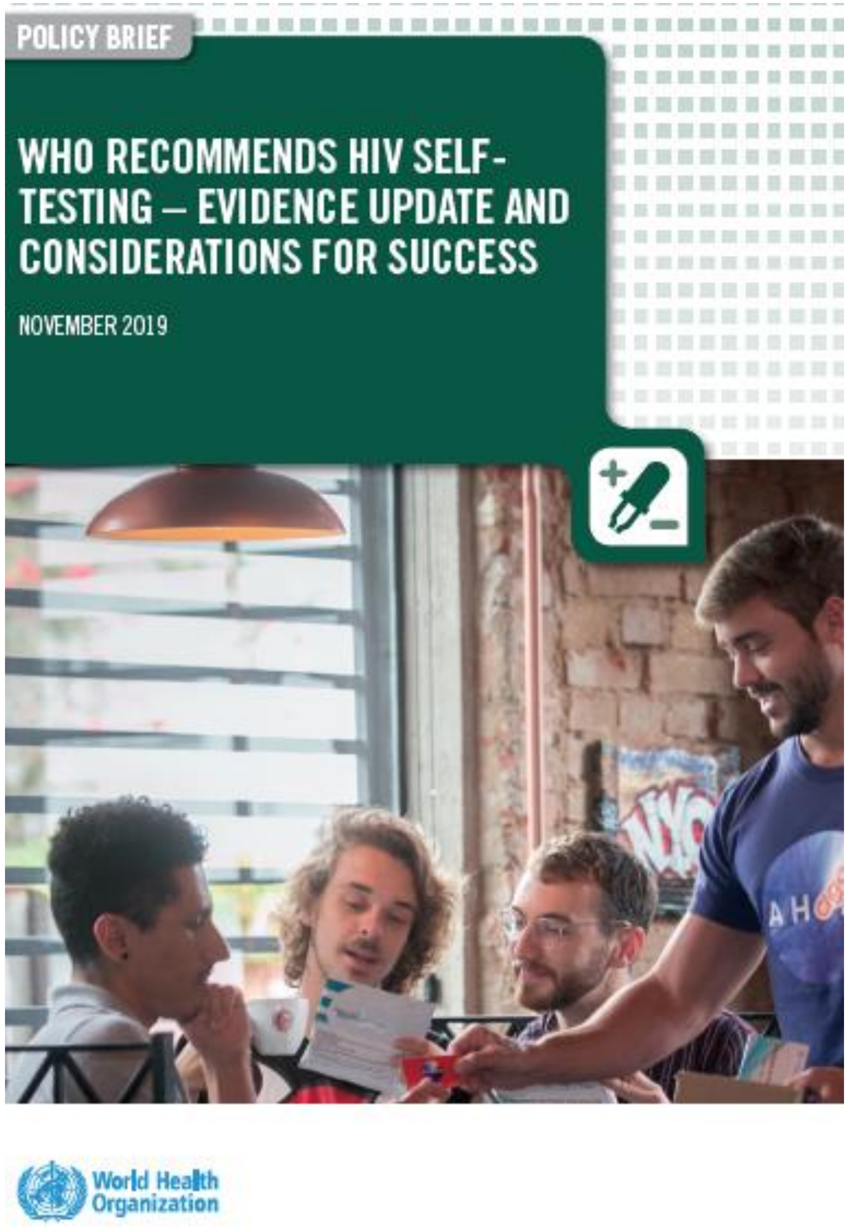

Community-based
Distribution during campaigns, at events, mobile outreach or home-based (door-to-door) distribution. Integration with existing community-based testing programmes can improve efficneicy and optimize resources. Community-led models are likely to be successful.

Facility-based
Distribution from facilities or other fixed sites for use later or within the facilities. Kits can be given to HIV-positive or HIV-negative clients for secondary distribution.

Order online and receive via mail
A range of online platforms such as websites, social media, dating apps, and other digital media can be used. HIVST kits can be provided for free, at a cost or with coupons/vouchers for reduced cost.

Secondary distribution
Includes secondary distribution to partners or peers including distribuiton by HIV-negtive and HIV-positive clients. Index clients can be given HIVST kits by providers at facilities.

Retail outlets, pharmacies and vending machines
Kits are typically provided at a cost to users but price can be reduced through public-private partnerships and distribution of coupons or vouchers.

Faith-based settings
Distribution from faith-based settings such as churches and mosques.

Workplace
Distribution to workers for testing themselves and/or for their partners. Consider sustainable models such as through public-private partnership and/or insurance packages to cover the cost.

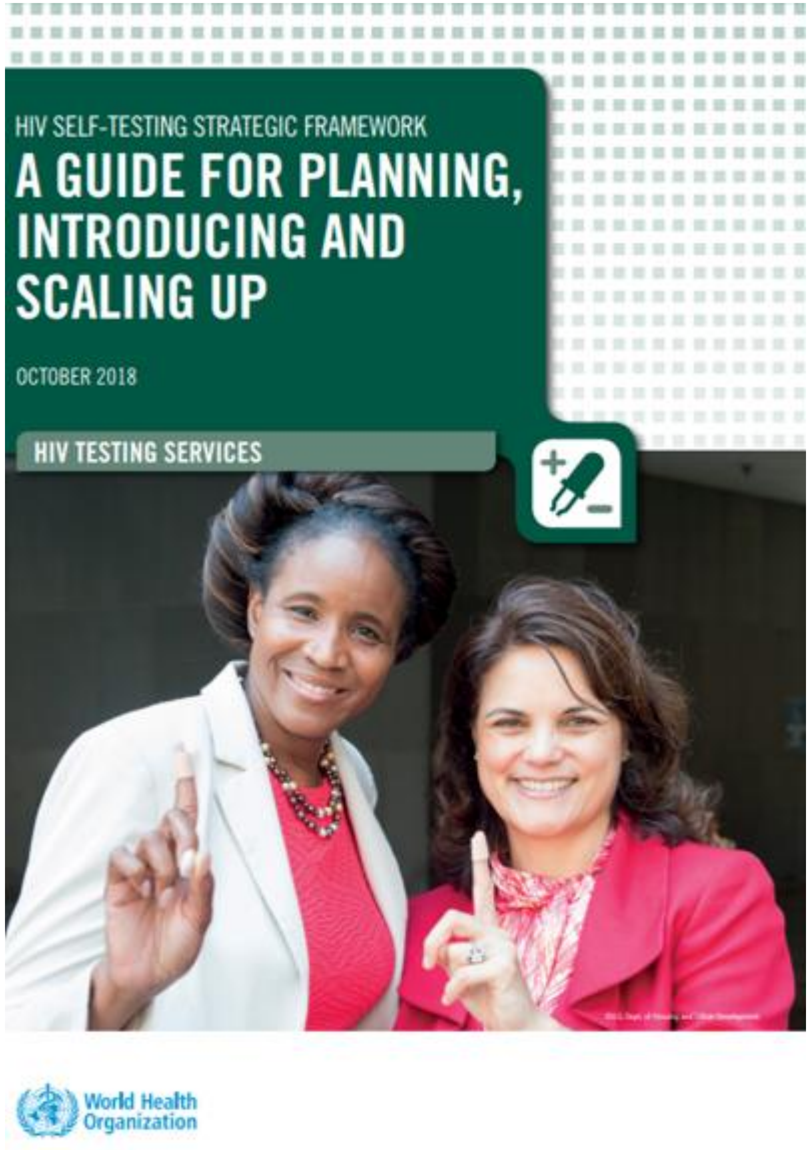

HIV self-testing and testing for triage

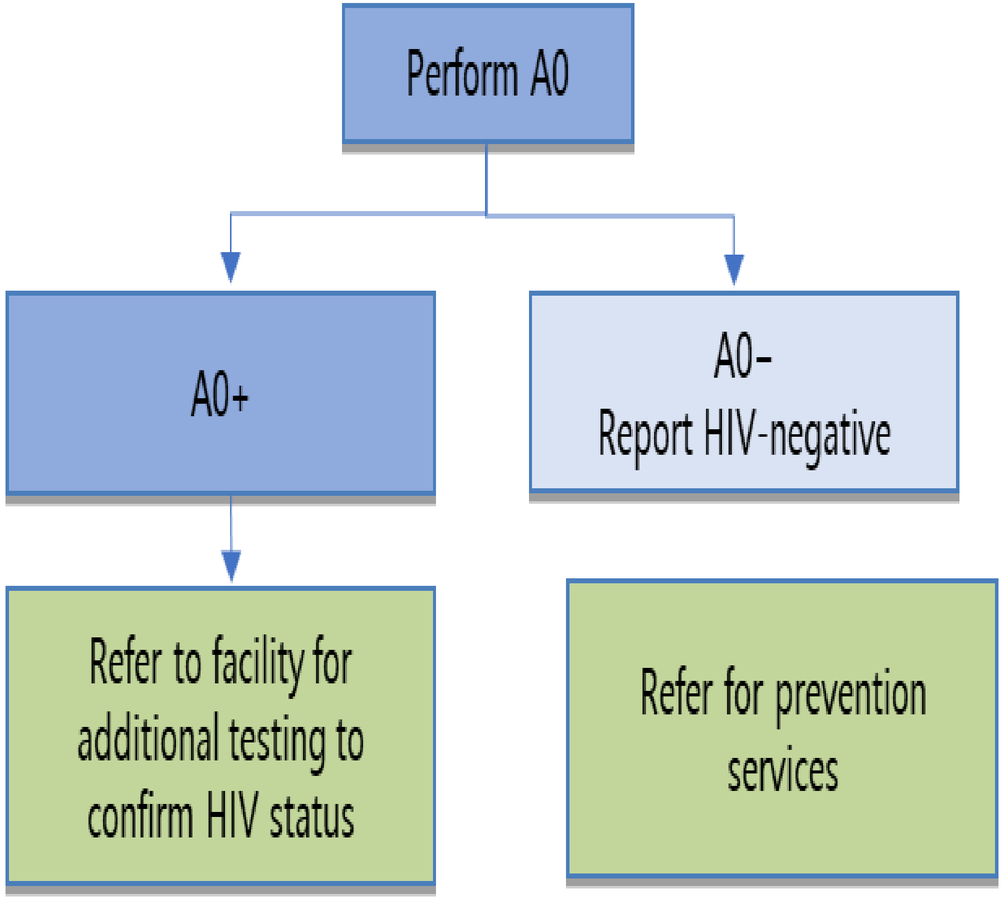

A0: Assay 0

- A trained provider (possibly a lay community health worker or the individual) conducts a single HIV RDT [Assay 0 (A0)]
- A0 does not replace Assay 1 of WHO testing strategy
- In case of a reactive A0 the person is linked by the testing provider to a facility where standard HIV testing algorithm is conducted
- In case of the person performing the test personally she/he has to contact community health worker or the health facility to confirm HIV status
- All A0 negative should receive HIV prevention services

World Health Organization

Variety of support tools for HIVST

1. In-person demonstration (one-on-one, with partners or in groups)
2. Demonstration video (including online links to videos)
3. Telephone hotline (can be integrated into existing national hotline services)
4. Short message service through telephone, Internet, social media
5. Educational information via radio, television, leaflets, brochures, the Internet, social media and applications for smartphones/tablets
6. Local information and resources, for example on counselling services, testing sites, treatment centres and where to access HIV prevention services like VMMC and PrEP.

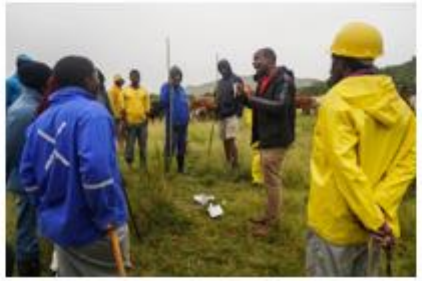

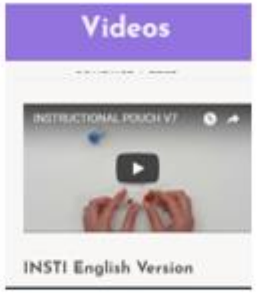

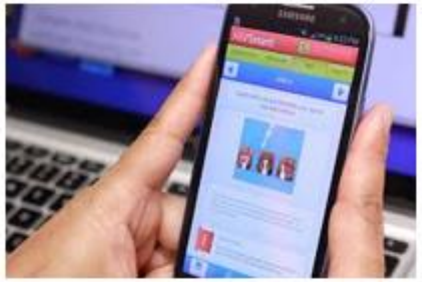

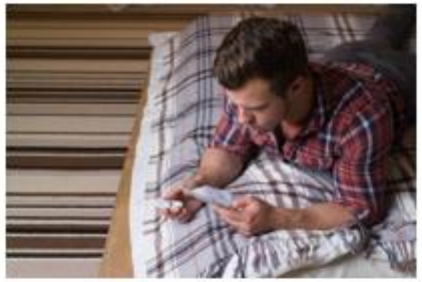

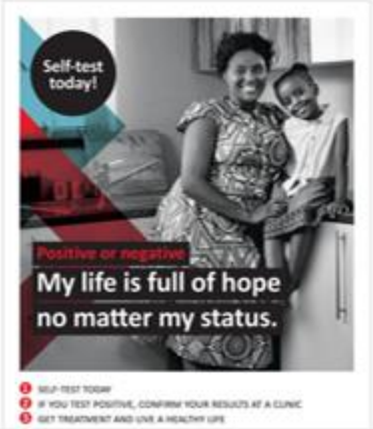

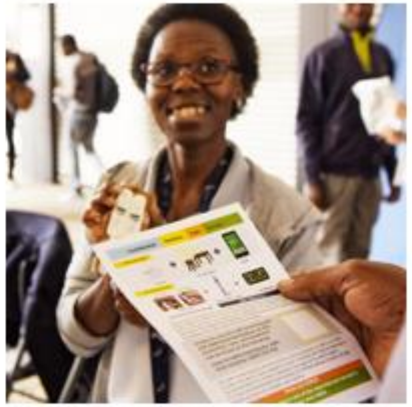

World Health Organization

Where to Begin with HIV Self-Testing

Know your epidemic & testing gap	Approaches		Considerations
Couples & Partners	Community-based (outreach, door-to-door)	Facility-based (PITC, drop-in centres)	Benefits & Risks to Populations
Men	VMMC programmes	Workplace programmes	Support tools
Key populations	Pharmacies & Kiosks	Integrated in KP Programmes	Linkage
Young people	Internet & Apps	Integrated in RHS & Contraceptive Services	Increased access
Other At risk populations (SDC, partners of PLHIV, migrants etc.)	Vending machines	Partner-delivered	Increased coverage

Where to Begin with HIV Self-Testing

Know your epidemic & testing gap	Approaches		Considerations
Couples & Partners	Community-based (outreach, door-to-door)	Facility-based (PITC, drop-in centres)	Benefits & Risks to Populations
Men	VMMC programmes	Workplace programmes	Support tools
Key populations	Pharmacies & Kiosks	Integrated in KP Programmes	Linkage
Young people	Internet & Apps	Integrated in RHS & Contraceptive Services	Increased access
Other At risk populations (SDC, partners of PLHIV, migrants etc.)	Vending machines	Partner-delivered	Increased coverage

HIVST projects

World Health Organization

Aviro digital stories for HIV testing in South Africa

Why get an HIV test?

Many people do not think they are at risk of getting HIV. Watch the videos below to learn about difference experiences and why getting tested is important for everyone.

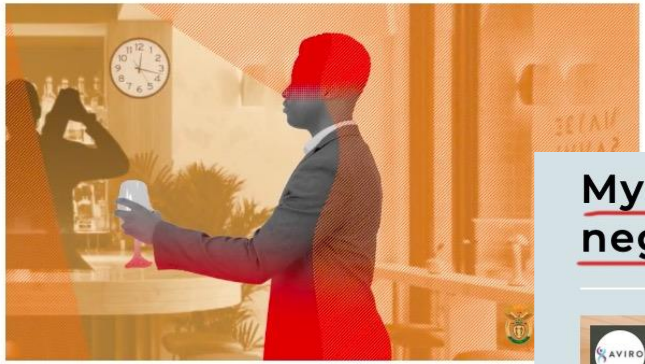

My HIV Test

Here we meet Lizette, Thabo, Valencia and the Khumalos. They all take us through their respective journeys and relationships. They let us in on their possible risks of getting HIV and why they decided to get tested.

My HIV test result is negative

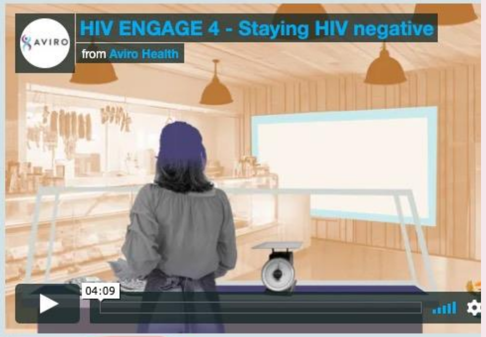

Staying Negative

Lizette continues to take us through her journey as a partner to Donnie, and how she is taking care of herself to ensure that she stays HIV negative while she is figuring out other aspects of her life and marriage.

My HIV test result is positive

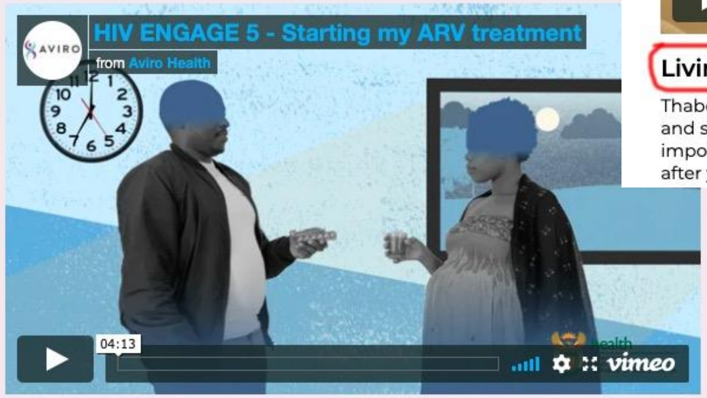

Starting my ARV Treatment

Valencia and Thabo take us through their journey on taking ARV treatment. Mr Khumalo also lets us in on how he is supporting his wife in taking her ARVs while he is taking his PrEP to protect each other and their beautiful family.

Living with HIV - what do you need to know

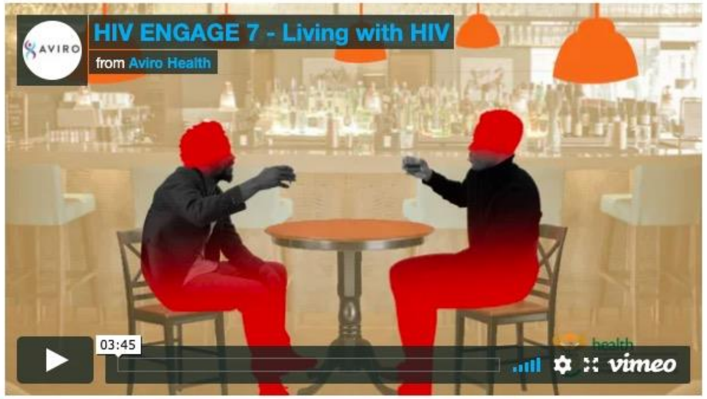

Living with HIV and Dealing with Stigma

Thabo lets us in on his experience with starting ARV treatment and stopping because he was doing well. His journey is an important lesson on staying adherent and consistently looking after your health and taking care of yourself.

Source: https://hivengage.co.za/hiv-content

World Health Organization

Aviro’s Ithaka portal promoting HIV self-testing in Africa

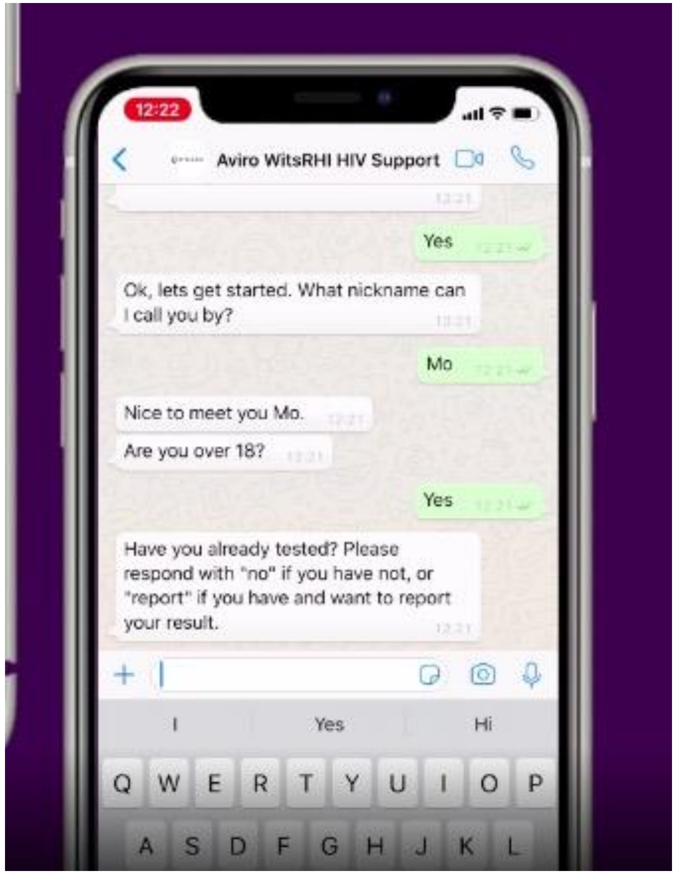

Features

Counseling Features:

NDOH approved content (text and video) for HIV counseling (RDT)
Oral and blood HIV self-testing support
Multi-language support
Linkage to Care support
Index testing referral support

Platform Features:

Tablet app (online/offline)
Whatsapp chatbot
Data free web application (PWA)
Digital Collection Form builder
Custom branding
POPI compliant (See Aviro **Privacy Policy and T&Cs**)
Data dashboards
Integration to existing data systems

Request a Demo

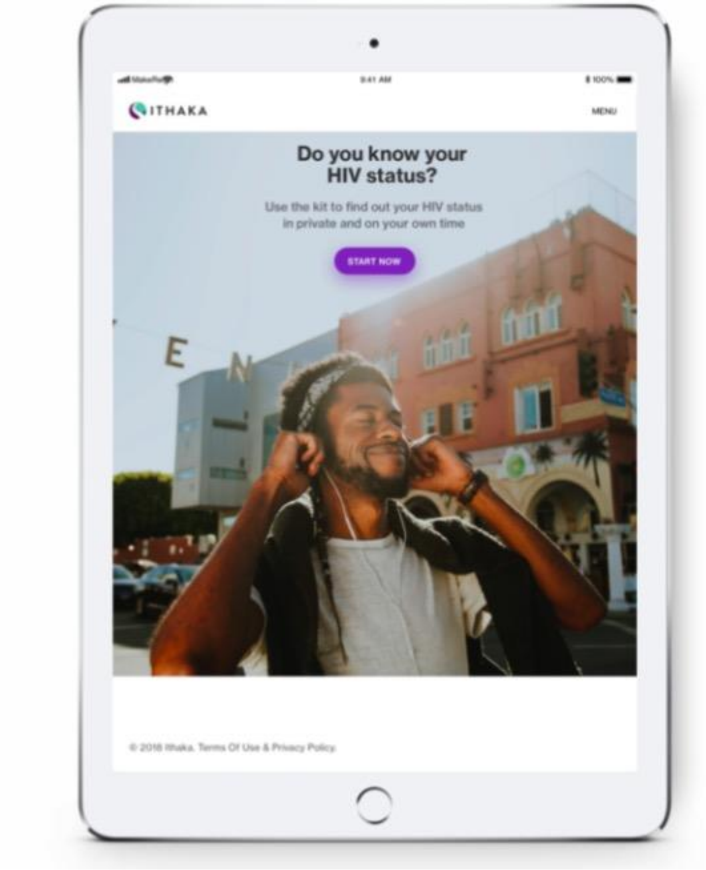

Source: https://www.avirohealth.com/ithaka/

World Health Organization

Online distribution and support of HIV self-testing in Manila

- Demonstration project implemented by LoveYourself (NGO) in Metro Manila area
- Clients assisted via a specially developed chat-box for delivery and testing process
- Volunteers provided assistance and counseling
- Condoms and lubricants also included in HIVST kit delivery
- Kits were meant to last six months, but allocated kits were used up within a month of implementation (14 Apr-14 May) due to lockdown
- **4,205** unique clients expressed interest; **2,259** were qualified; **1,689** were successfully delivered; **1,133** reported their results
- **93** were validated reactive (8% reactivity rate); **55** linked to treatment

Source: Love Yourself Inc. Acceptability and feasibility of HIV self—screening among MSM and transgender women in Metropolitan Manila. Technical Report, 2020.

loveyourself

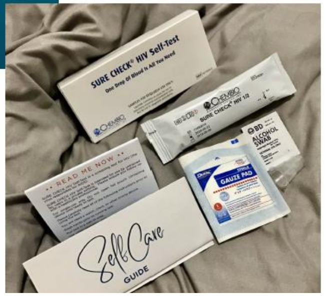

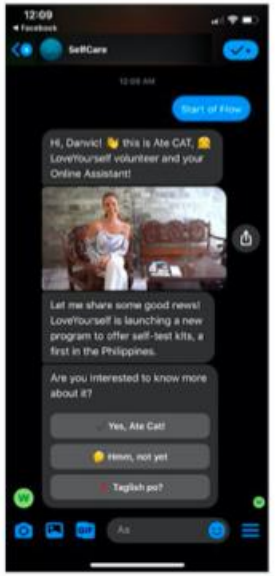

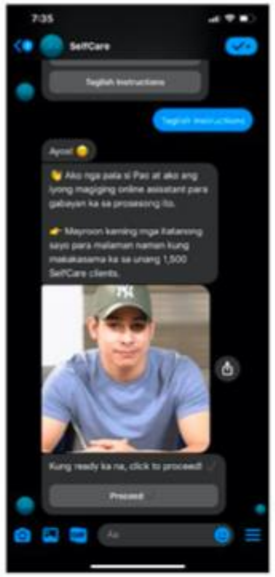

Online approach for HIV self-testing distribution in Sri Lanka

- The National STD/AIDS Control Programme is distributing HIV self-testing kits among key population through its network of NGOs and CBOs
- Key population persons can access the website in three different languages
- Interested persons has to fill the required information and give consent
- HIV self-test kit will be sent to the persons address via courier
- Support available via online chat

Sources:
https://www.aidscontrol.gov.lk/index.php?lang=en;
https://know4sure.lk/

National STD/AIDS Control Programme - Sri Lanka
Ministry of Health, Nutrition & Indigenous Medicine

KNOW 4SURE

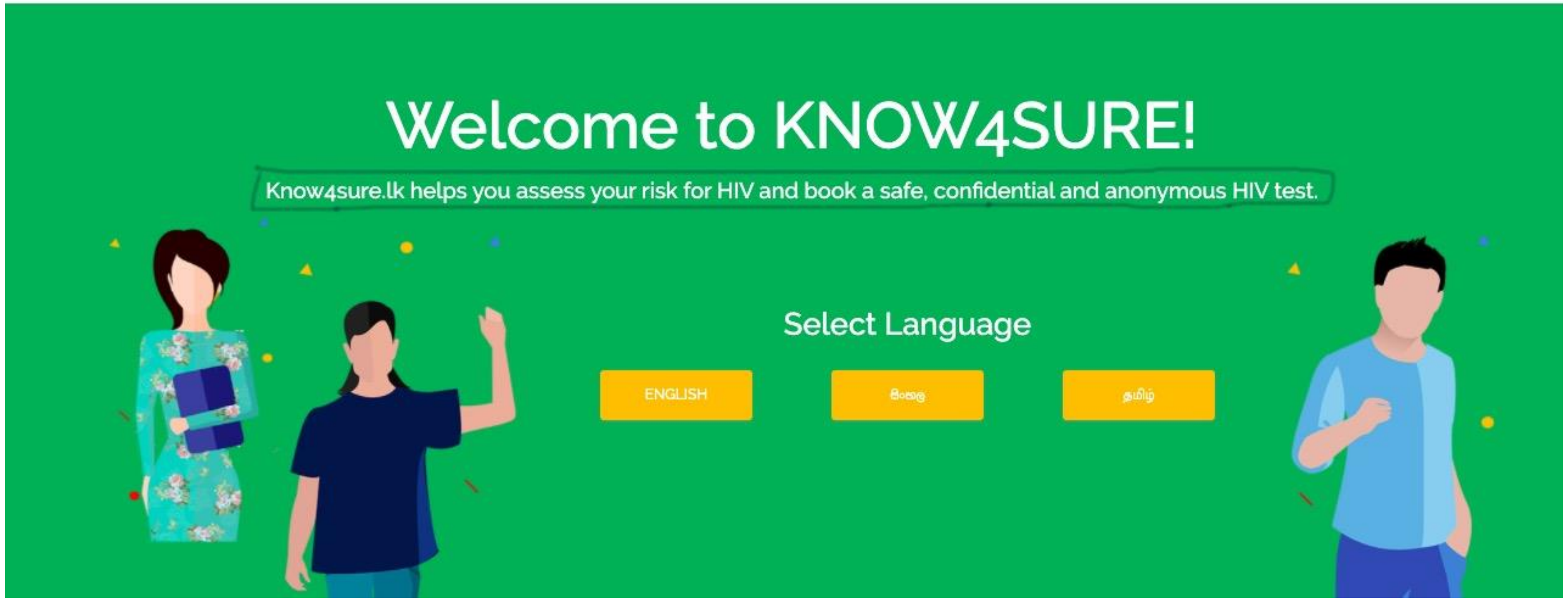

World Health Organization

Online distribution of HIV self-testing in Malaysia

- MSM, TGs and sex workers and their partners will be offered HIVST package via **Jomtest website**
- Interest participants will have to complete the following steps:
    1. Register
    2. Update address
    3. Fill a pre survey online form
    4. Order test kit
    5. Wait for response
    6. Self-test kit delivery
    7. Self-testing
    8. Upload result in the website
    9. Answer post survey
    10. Complete
- Support available via Malaysian AIDS Council and Kuala Lumpur AIDS Support Services Society (KLASS)

JOM TEST
Fast . Accurate . Confidential

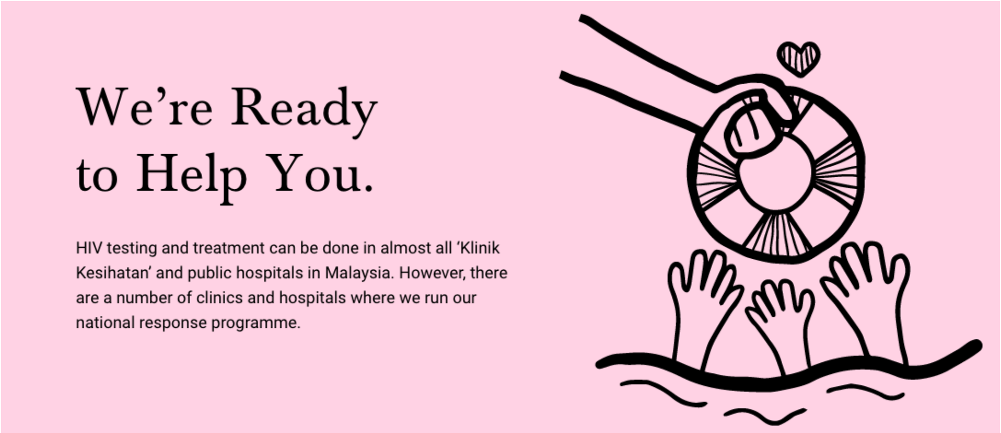

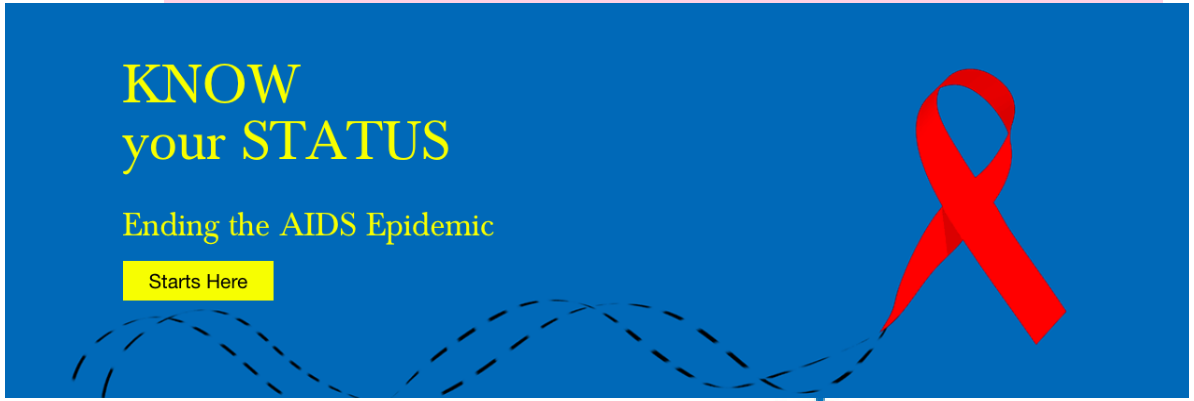

World Health Organization

HIV self-testing kits distribution via FB messenger and WhatsApp in Pakistan

- HIVST kits to be distributed among MSM and TGs via two approaches
- First will be peer led community based approach and other will be via social media platforms such as FB messenger and WhatsApp
- Study sites: one urban center and other a rural town
- Key outcomes:
  - Proportion of initial reactive cases who proceeded for confirmatory test.
  - Percentage of confirmed HIV positive linked to ART initiation.
  - Identifying which approach worked better in distributing HIVST kits to KPs.
- Timeline October to December 2020
- Implementers: WHO, local CBOs and AIDS Control Programme

World Health Organization

Self-screening South Africa

- HIV Self-Screening is funded by Unitaid in South Africa to support the National Department of Health (NDoH) to increase access to HIV testing services
- To ensure widespread access to these kits, request a HIV self-screening kit for free from selected independent pharmacies
- The funder of this project has covered all the costs for this initiative to increase access on behalf of the NDoH

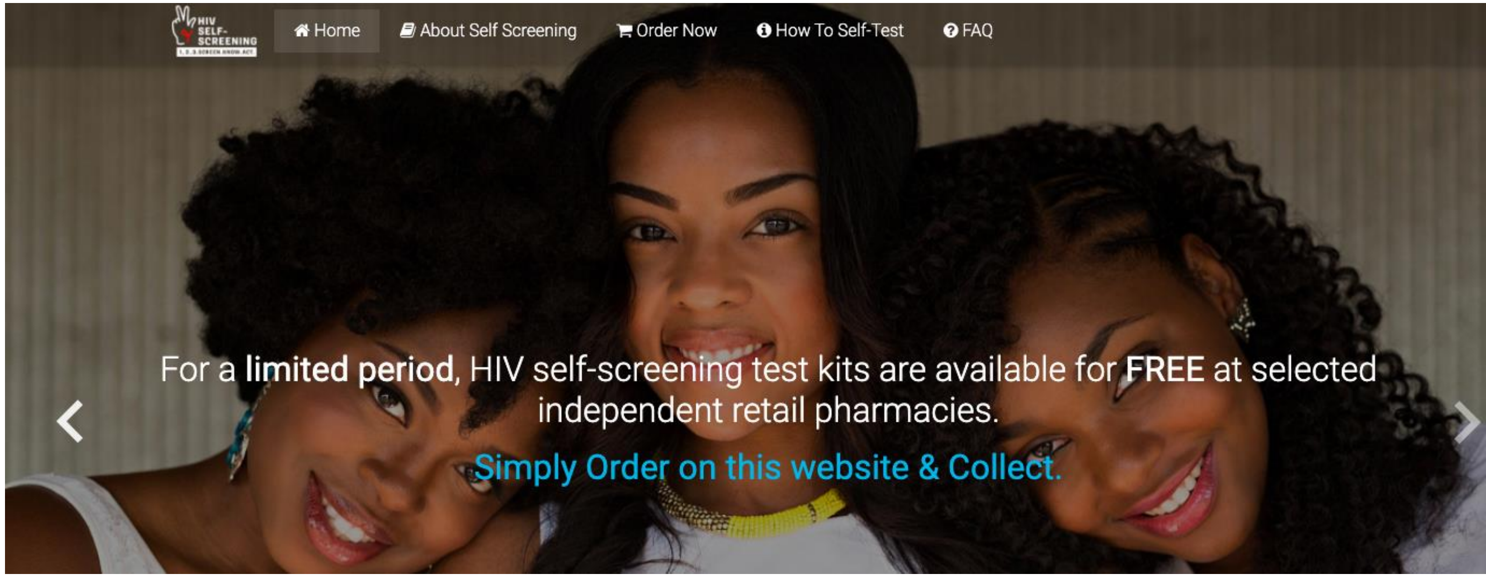

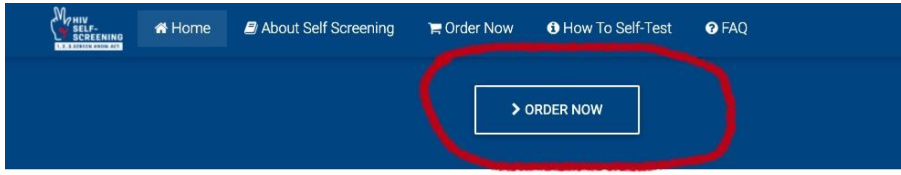

How to use the OraQuick® HIV Self-Screening Kit

You must follow the test directions carefully to get an accurate result. Do not eat or drink for at least 15 minutes before you start the test or use mouth cleaning products 30 minutes before you start the test.

Simply follow this step-by-step guide on how to take the test

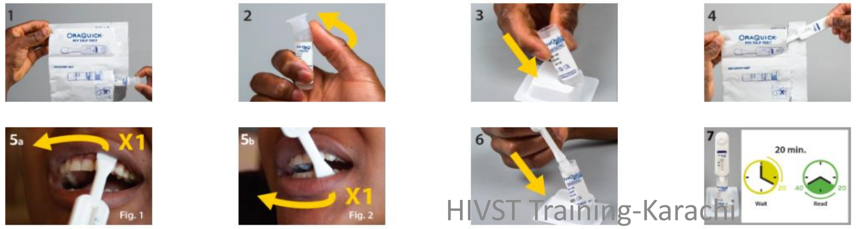

Benefits of OraQuick® HIV Self-Screening kit

- Conduct a pain free swap of your gums
- Takes 20 minutes to get the result
- Easy to follow instructions
- High level of accuracy
- Screens for the earliest HIV antibodies

HIVST products with WHO prequalification

Test (manufacturer)	Type
**INSTI® HIV Self Test ****<br>(bioLytical Lab., Canada)	Blood
**Mylan HIV Self Test**	Blood
**OraQuick® HIV Self Test**<br>(OraSure Technologies, USA)	Oral
**SURE CHECK® HIV Self Test**<br>(Chembio Diagnostic Systems Inc., USA)	Blood

Global Fund HIVST pricing
US$ 2-3.10

More information available from Global Fund PSM/Sourcing team

Latest list of WHO prequalified products: https://www.who.int/diagnostics_laboratory/evaluations/PQ_list/en/

Community led and lay provider testing-Viet Nam (2017-2018)

Nguyen TTV et al. *Journal of the International AIDS Society* 2019, **22**(S3):e25301
http://onlinelibrary.wiley.com/doi/10.1002/jia2.25301/full | https://doi.org/10.1002/jia2.25301

JIAS JOURNAL OF THE INTERNATIONAL AIDS SOCIETY

RESEARCH ARTICLE

Community-led HIV testing services including HIV self-testing and assisted partner notification services in Vietnam: lessons from a pilot study in a concentrated epidemic setting

Van Thi Thuy Nguyen[1§], Huong TT Phan[2], Masaya Kato[1], Quang-Thong Nguyen[3], Kim A Le Ai[4], Son H Vo[2], Duong C Thanh[5], Rachel C Baggaley[6] and Cheryl C Johnson[6,7]

[§]**Corresponding author:** Van Thi Thuy Nguyen, WHO, 304 Kim Ma, Ba Dinh District, Hanoi, Vietnam. Tel: +84 2438500314. (nguyenva@who.int)

Indicator	Number (percentage)
KP persons tested	3978
First time tester	66.7%
Tested by lay provider	3086 (77.6%)
Initial reactive	245 (6.2%)
Confirmed HIV positive	231 (94.2%)
On ART treatment	215/231 (93.1%)

- Community led approach for key population is effective and feasible
- Particularly for reaching first time testers
- Lay providers played a key role in increasing the number of those tested

World Health Organization

Dual HIV/syphilis rapid diagnostic in ante natal care

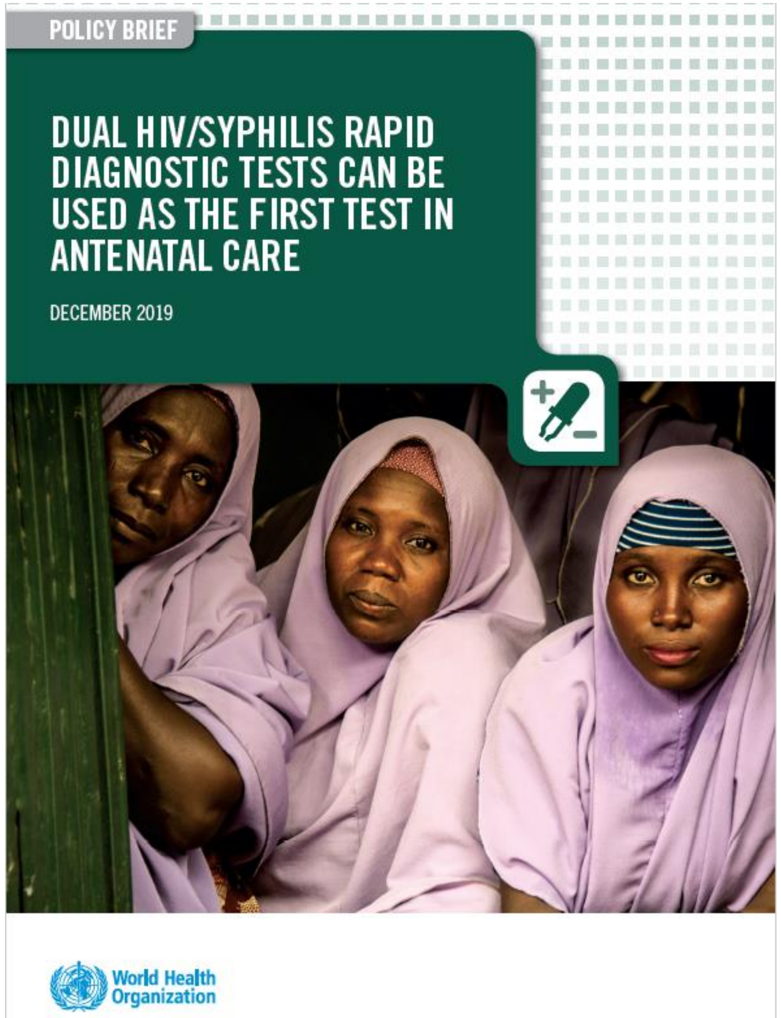

- **WHO recommends that pregnant women should receive testing for HIV, syphilis and hepatitis B (HBSAg) at least once during pregnancy**
- **Dual HIV/syphilis testing can help identify infections among pregnant FSWs**
- Should be conducted preferably in the first trimester
- These tests are cheaper compared to standard testing in ANC

World Health Organization

Optimal maternal retesting time points

- **Key population**
  - FSWs in serodiscordant relationships (one partner HIV positive and the other HIV negative)
- **In high burden settings**
  - All pregnant women with an unknown HIV status or HIV negative should be tested
  - Should be retested in late pregnancy (3rd trimester)
  - At any health facility visit six months postpartum

Testing strategy for dual HIV/syphilis in ANC

- Countries introducing dual HIV/syphilis RDT as the first test in ANC will need to revise their HIV testing strategy for pregnant women
- Dual HIV/syphilis should not be used for:
  - Women with HIV taking ARTs
  - Women already diagnosed with and treated for syphilis during their current pregnancy
  - Retesting for HIV

World Health Organization

Important considerations for dual HIV/syphilis RDTs

- Countries should first verify that the new test works in combination with other two HIV tests in the algorithm
- <u>Programmes should consider other settings e.g. outreach to key population at risk of HIV and syphilis</u>
- HIV/syphilis use can lead to diagnosis of more syphilis cases
  - Programmes should prepare their selves for addition treatment procurement (benzathin penicillin)
- Training of staff including testers, implementers, procurement specialists will be necessary on all aspects of HIV/syphilis RDTs

WHO recommended testing strategy for dual HIV/syphilis detection in ANC settings

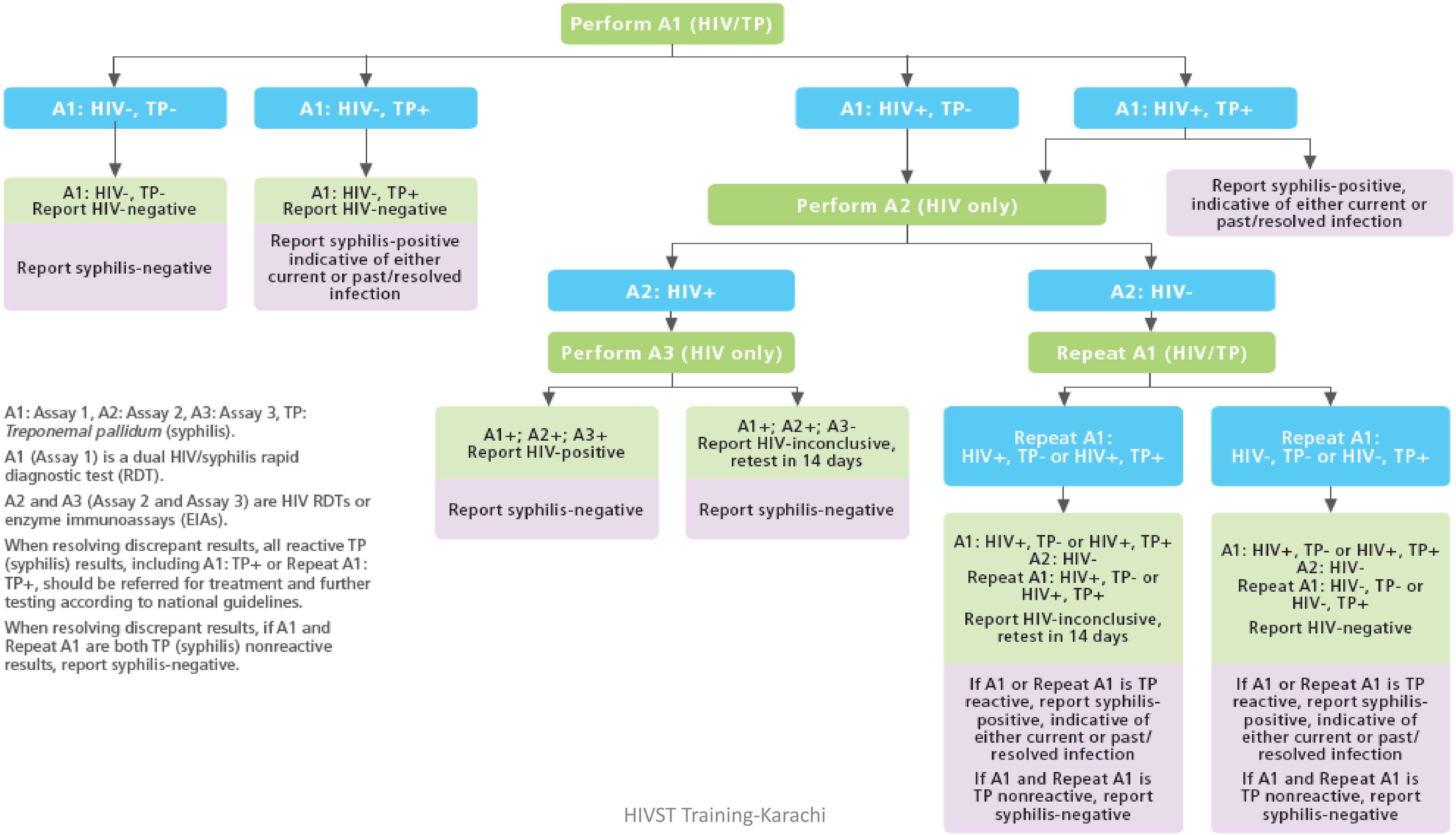

A1: Assay 1, A2: Assay 2, A3: Assay 3, TP: *Treponemal pallidum* (syphilis).

A1 (Assay 1) is a dual HIV/syphilis rapid diagnostic test (RDT).

A2 and A3 (Assay 2 and Assay 3) are HIV RDTs or enzyme immunoassays (EIAs).

When resolving discrepant results, all reactive TP (syphilis) results, including A1: TP+ or Repeat A1: TP+, should be referred for treatment and further testing according to national guidelines.

When resolving discrepant results, if A1 and Repeat A1 are both TP (syphilis) nonreactive results, report syphilis-negative.

Summarizing

- WHO supports implementation and scale up of a strategic mix of evidence based HTS approaches in both facility and community settings, to reach those who have not been tested
- All those tested should have access to effective linkage, appropriate prevention, treatment and care services
- WHO encourages integration of HIV testing and other relevant services

Access the full guidelines on the WHO HTS APP!

- Search ‘WHO HTS Info’ wherever you get Apps
- Notifications when new content is available
- Search, save, send
- Country HTS data in one place w/ guidelines
- Language updates: French on the way!
- Available online and off
- Videos coming for 2020

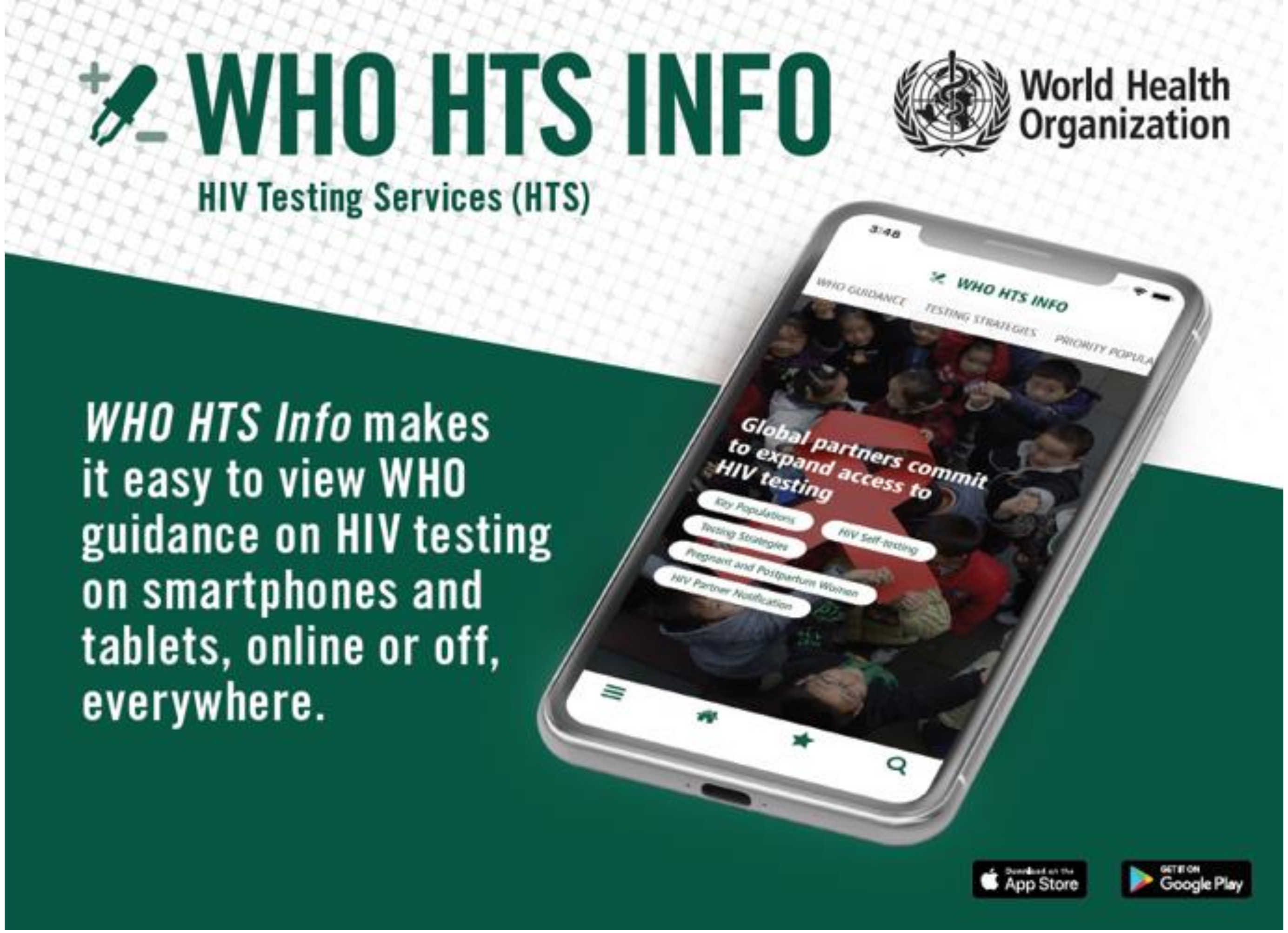

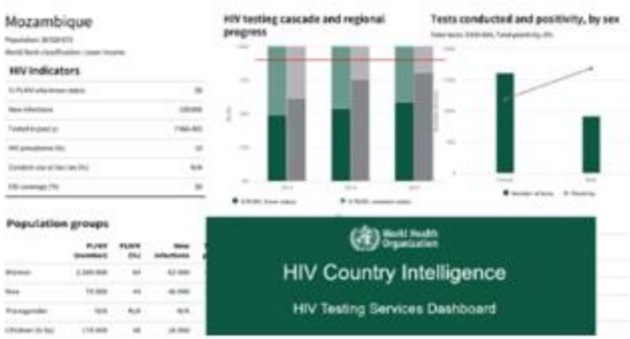

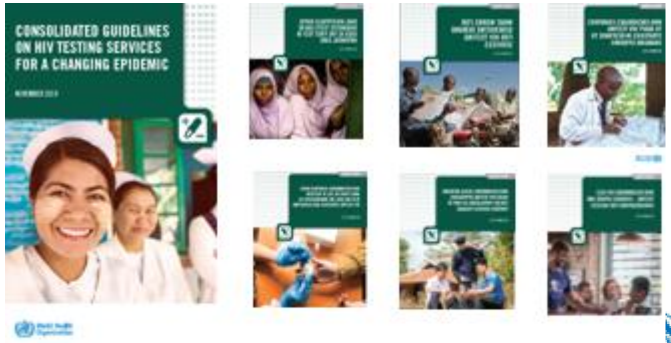

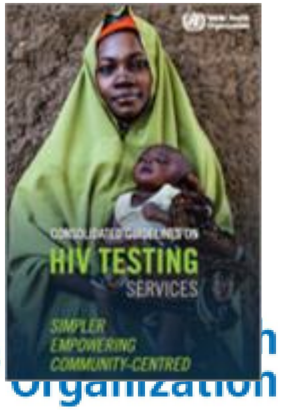

For more information, contact:

World Health Organization
Department of HIV
20, avenue Appia
1211 Geneva 27
Switzerland

E-mail: hiv-aids@who.int

http://www.who.int/hiv/pub/vct/en/

ISBN 978 92 4 155058 1

World Health Organization
