# Supplementary material for: Acceptability and feasibility of HIV self-testing among transgender people in Larkana, Pakistan: Results from a pilot project
Source: PLoS One. 2022 Jul 8;17(7):e0270857. doi: 10.1371/journal.pone.0270857 (PMC9269381; doi:10.1371/journal.pone.0270857)
Supplement: S1 File — (ZIP) [file pone.0270857.s001.zip › Supporting files/Indepth interviews KPs_FINAL.pdf]

ایم ایس ایم اور ٹی جی کے ساتھ اِن ڈیپتھ انٹرویو

In Depth Interview with KPs

اسٹڈی کا نام: ایچ آئی وی سیلف ٹیسٹنگ کٹ تقسیم/ بانٹنے کا پروجیکٹ

Demostration project to determine acceptable distribution model

for HIV self-testing kits among key population in Pakistan

تعارف:

آپ کا ایچ آئی وی سیلف ٹیسٹنگ کٹ پروجیکٹ میں شامل ہونے کا شکریہ۔ اور اس انٹرویو کے لئے راضی ہونے پر بھی آپ کے شکریہ۔ اگر آپ اجازت دیں تو میں آپ سے ایچ آئی وی سیلف ٹیسٹنگ پروجیکٹ میں شامل ہونے کے بارے میں کچھ سوالات کرونگا۔

سوال نمبر۱: آپ کا اِس پروجیکٹ میں شامل ہونے کا تجربہ کیسا رہا؟

سوال نمبر۲: کیا آپ کے خیال میں ایم ایس ایم اور ٹی جی پاکستان میں ایچ آئی وی سیلف ٹیسٹ خود سے کر سکتے ہیں؟ آپ کے خیال میں اس میں کیا مشکلات پیش آسکتی ہیں اور اِن مشکلات کو کیسے ٹھیک کیا جا سکتا ہے؟

سوال نمبر۳: آپ کے خیال میں ایچ آئی وی سیلف ٹیسٹنگ کٹ کی پاپولیشن (key population) میں کیسے تقسیم/ بانٹی جا سکتی ہیں؟

PDF created with pdfFactory Pro trial version www.pdffactory.com
