# Supplementary material for: Acceptability and feasibility of HIV self-testing among transgender people in Larkana, Pakistan: Results from a pilot project
Source: PLoS One. 2022 Jul 8;17(7):e0270857. doi: 10.1371/journal.pone.0270857 (PMC9269381; doi:10.1371/journal.pone.0270857)
Supplement: S1 File — (ZIP) [file pone.0270857.s001.zip › Supporting files/Information sheet_FINAL.pdf]

انفارمیشن شیٹ۔ اسٹڈی میں شامل حضرات کے لئے

Information Sheet

اسٹڈی کا نام: ایچ آئی وی سیلف ٹیسٹنگ کٹ تقسیم/ بانٹنے کا پروجیکٹ

Demostration project to determine acceptable distribution model for HIV self-testing kits among key population in Pakistan

آپ کا بہت شکریہ جو آپ نے میری بات سننے کیلئے وقت نکالا۔ ہم مخصوص پاپولیشن میں ایچ آئی وی سیلف ٹیسٹنگ کٹ بانٹنے کا ایک پروجیکٹ شروع کرنے جارہے ہیں۔ میں آپ کو اِس پروجیکٹ کے بارے میں کچھ معلومات دینا چاہتا/ چاہتی ہوں۔ یہ معلومات آپ کو اس پروجیکٹ میں یہ فیصلہ کرنے میں مدد کرے گی کہ آپ اس میں شامل ہونا چاہتے ہیں یا نہیں۔ آپ اس کے بارے میں دوسروں سے بھی بات چیت کر سکتے ہیں۔

مزید معلومات کے لئے آپ مجھ سے یا میرے سپروائزر سے بھی بات چیت کر سکتے ہیں۔

پروجیکٹ کا مقصد

اس پروجیکٹ کا مقصد ایم ایس ایم اور ٹی جی کمیونٹی میں ایچ آئی وی ٹیسٹنگ کی اہمیت کو اجاگر کرنا ہے۔ اس کے ساتھ ساتھ اس بات کی بھی نشاندہی کرنا ہے کہ احتیاط اور علاج کتنا ضروری ہے۔ اور ہم یہ بھی جاننا چاہتے ہیں کہ ایم ایس ایم اور ٹی جی کمیونٹی میں ایچ آئی وی سیلف ٹیسٹنگ کٹ تقسیم کرنے کیلئے کونسا طریقہ زیادہ بہتر ہے۔

پروجیکٹ کے فائدے

پاکستان میں ایچ آئی وی بہت تیزی سے پھیل رہا ہے خصوصاً اُن مرد حضرات میں جو دوسرے مرد کے ساتھ سیکس کرتے ہیں۔ اِن افراد میں ایچ آئی وی کا ٹیسٹ کرنا بہت ضروری ہے۔ تاکہ ان میں سے اُن مردوں اور ہیجڑوں کو جن میں ایچ آئی وی موجود ہے اِن کو ٹریٹمنٹ پر جلد سے جلد ڈالا جا سکے۔ آپ کو شائد معلوم نہیں ہوگا کہ ایچ آئی وی کا علاج اور اِس کی دوائیاں آپ کے شہر میں مفت میں ملتی ہیں۔ پاکستان کے ایچ آئی وی کے ایکسپرٹس کا یہ خیال ہے کہ ایچ آئی وی سیلف ٹیسٹنگ ایک نہایت ہی آسان طریقہ ہے۔ ایچ آئی وی کا ٹسیٹ کرنے کا خصوصاً اُن حضرات میں جن میں ایچ آئی وی انفکیشن ہونے کا امکان زیادہ ہے کیونکہ ایچ آئی وی سیلف ٹیسٹنگ کوئی بھی شخص اپنی سہولت اور رازداری کے ساتھ جب چاہے کر سکتا/ کر سکتی ہے۔

کونسے افراد اس پروجیکٹ کا حصہ نہیں ہو سکتے

۱۔ جو ایچ آئی وی پازیٹو ہیں۔

۲۔ اگر آپ ایچ آئی وی کی دوائیاں (اے آر ٹی۔ARTs) لے رہے ہیں۔

PDF created with pdfFactory Pro trial version www.pdffactory.com

۳۔ اگر آپ کی عمر 18 سال سے کم ہے۔

ایچ آئی وی سیلف ٹیسٹنگ کا طریقہ

اگر آپ اِس پروجیکٹ کا حصہ بننے پر رضامند ہیں تو آپ کو ایک ایچ آئی وی سیلف ٹیسٹنگ کٹ درنگا/ دونگی۔ آپ یہ سیلف ٹیسٹنگ کٹ اپنے ساتھ لے جاسکتے ہیں اور اپنی سہولت سے جب چاہیں یہ سیلف ٹیسٹنگ کٹ استعمال کر سکتے ہیں۔ اگر آپ کو اس کے استعمال میں کسی قسم کی مدد چاہیئے وہ میں اور میری ٹیم What'sapp یا فیس بک Messenger ویڈیو کے ذریعے دے سکتے ہیں۔ اگر آپ ٹیسٹ کٹ کو استعمال کرنے می میری یا کسی اور ٹرینڈ ورکر کی مدد چاہتے ہیں تو وہ بھی ممکن ہے۔

سیلف ٹیسٹنگ کٹ کو استعمال کرنے کا طریقہ کار:

۱۔ اِس سیلف ٹیسٹ کو کرنے میں تقریباً 20 منٹ لگتے ہیں۔

۲۔ اِس ٹیسٹ میں خون کا کوئی استعمال نہیں ہے۔

۳۔ یہ ٹیسٹ مکمل طور پر راز میں رہ سکتا ہے۔

۴۔ آپ اِس نمبر پر فون بھی کر سکتے ہیں ________________

۵۔ سیلف ٹیسٹنگ کٹ کے استعمال سے پہلے 30 منٹ پلیز کچھ کھائیں اور پئیں نہیں۔ ٹوتھ پیسٹ یا ماؤتھ واش بھی استعمال نہ کریں۔

۶۔ اگر آپ کے منہ/ جبڑوں میں کوئی ڈینچر یا بریس لگا ہوا ہے تو اس کو نکال دیں۔

۷۔ کوئی ایسی جگہ ڈھونڈ کر بیٹھیں جہاں پر آپ کم از کم 20 منٹ تک کسی کے ڈسٹرب کئے بغیر بیٹھ سکتے ہیں۔

۸۔ کوئی چھوٹی میز یا اسٹول اپنے سامنے رکھ لیں۔

۹۔ اگر آپ پڑھ سکتے ہیں تو پلیز اس کٹ کے استعمال کا طریقہ کار ایک بار پڑھ لیں۔

۱۰۔ جس پیکٹ میں ٹیوب ہے اس کو کھول لیں۔

۱۱۔ ٹیوب کو ٹیوب ہولڈر میں فکس کر لیں۔

۱۲۔ آرام سے ٹیوب کا کیپ نکال لیں۔

۱۳۔ اب ٹیسٹ اسٹک کو نکال لیں اس اسٹک میں جو پیڈ ہے اُس کو اپنی انگلیوں سے مت چھوئیے گا۔

۱۴۔ پیڈ کو اپنے اوپر والے جبڑوں میں ایک بار گھمائیں اور ایک بار نیچے والے جبڑوں میں گھمائیں۔ یہ بہت ضروری ہے کہ آپ دونوں جبڑوں میں صرف ایک بار ہی گھمائیں گے۔ آپ پیڈ کی کوئی بھی سائیڈ استعمال کر سکتے ہیں۔

۱۵۔ ٹیسٹ اِسٹک کو ٹیسٹ ٹیوب میں ڈال لیں۔ پہلے پیڈ والی سائیڈ ڈالیں۔

۱۶۔ ٹیسٹ ٹیوب کو ابھی باہر نہیں نکالیئے گا۔

۱۷۔ وقت نوٹ کر لیں اور 20 منٹ انتظار کریں۔

۱۸۔ ٹیسٹ اِسٹک میں گلابی کلر آئے گا جو کہ بالکل نارمل ہے۔

PDF created with pdfFactory Pro trial version www.pdffactory.com

۱۹۔ 20 منٹ انتظار کرنا ہے۔

۲۰۔ 40 منٹ سے زیادہ انتظار نہیں کرنا ہے۔ کیونکہ 40 منٹ کے بعد اس ٹیسٹ کا رزلٹ قابل اعتماد نہیں رہ سکتا۔

۲۱۔ اگر C (کنٹرول) کے برابر ایک لکیر اُبھر آئی ہے تو اِس کا مطلب ہے کہ آپ HIV نیگیٹیو ہیں۔

۲۲۔ اگر T کے برابر والی لکیر اُبھر جاتی ہے ہلکی سی بھی اس کا مطلب ہے کہ آپ کو HIV انفیکشن ہے۔

۲۳۔ اگر کوئی بھی لکیر اُبھر کر نہیں آئی اس کا مطلب ہوگا کہ ٹیسٹ صحیح طریقے سے نہیں کیا گیا۔

۲۴۔ اگر دو لکیریں آتی ہیں تو اس کا مطلب ہے کہ HIV کا کنفر میٹری ٹیسٹ کرانا بہت ضروری ہے۔ وہ ٹیسٹ HIV ٹیسٹنگ سینٹر سے فری میں ہوگا۔ آپ مجھے کال کریں اور میں آپ کے ساتھ ٹیسٹ کروانے کے لئے چلونگا/ چلوں گی۔

۲۵۔ استعمال شدہ کٹ کو تھیلی میں ڈال کر کوڑے دان یں پھینک دیں۔

۲۶۔ اگر آپ سمجھتے ہیں کہ آپ HIV کے رسک پر ہیں تو آپ کو چاہئے کہ اپنا HIV کا ٹیسٹ تین مہینے بعد دوبارہ کروائیں۔

اس ٹیسٹ کا یوٹیوب لنک

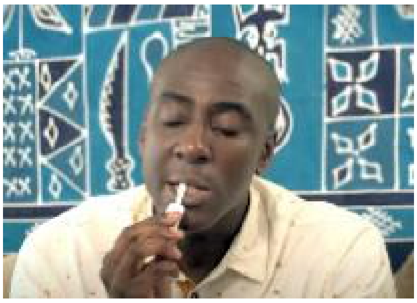

https://www.youtube.com/watch?v=dllddul79ic

کیا میں اس پروجیکٹ سے کسی بھی وقت نکل سکتا ہوں؟

جی ہاں، آپ جب چاہیں اِس پروجیٹ سے نکال سکتے ہیں۔ اس پر کوئی پابندی اور جرمانہ نہیں ہے۔

اگر میں اِس پروجیکٹ میں شامل ہونا چاہوں تو اُس کے لئے مجھے کیا کرنا ہوگا؟

۱۔ آپ کو یہ انفارمیشن شیٹ دی جائے گی۔

۲۔ آپ سے ایک اجازت نامہ لیا جائے گا۔

۳۔ آپ کو اپنا HIV سیلف ٹیسٹ کا رزلٹ مجھ سے شیئر کرنا ہوگا۔

۴۔ اگر آپ کا سیلف ٹیسٹ کٹ سے کیا گیا ٹیسٹ مثبت ہوا تو میں آپ قریبی ٹیسٹنگ سینٹر کنفرمیشن کیلئے لے کر جاؤں گا/ جاؤنگی جہاں پر کنفرمیشن کے بعد آپ کا معائنہ ہوگا اور اگر ڈاکٹر صاحب مناسب سمجھیں گے تو آپ کو HIV کی دوائیاں (ART) شروع کی جائیں گی۔

۵۔ اگر آپ کا سیلف ٹیسٹنگ کا رزلٹ نیگیٹیو ہوگا اس صورت میں میں آپ کو احتیاط کے بارے میں کچھ معلومات دونگا/ دونگی اور آپ کہاں سے مفت کنڈوم اور لبریکینٹ حاصل کرسکتے ہیں۔

PDF created with pdfFactory Pro trial version www.pdffactory.com

پروجیکٹ میں شامل ہونے کا دورانیہ

۱۔ اس کا دارومدار اس بات پر ہوگا کہ آپ اپنا سیلف ٹیسٹ کب کرتے ہیں۔

۲۔ اگر سیلف ٹیسٹ کٹ لینے کے ایک ہفتے بعد بھی آپ اپنا ٹیسٹ نہیں کرتے ہیں تو میں آپ کو فون کر کے یاد کرواؤں گا/کرواؤں گی۔

۳۔ اپنا سیلف ٹیسٹ کرنے کے بعد اور اس کا رزلٹ مجھ سے شیئر کرنے کے بعد میں آپ کو احتیاطی تدابیر کے بارے میں بتاؤں گا/گی اُس کے بعد میں آپ کا ایک انٹرویو کروں گا/کروں گی جو 20-15 منٹ میں پورا ہو جائے گا۔ یہ انٹرویو فون پر بھی ہوسکتا ہے۔

اس پروجیکٹ میں شامل ہونے کے فوائد

آپ کو فری میں ایک سیلف ٹیسٹ کٹ ملے گی جس سے آپ اپنا ایچ آئی وی کا اسٹیٹس اپنی سہولت سے چیک کر سکتے ہیں۔

اس پروجیکٹ میں شامل ہونے کے رسک

۱۔ اس پروجیکٹ می شامل ہونے کی وجہ سے ہم آپ سے کچھ ایسے سوالات کریں گے جن کا تعلق جنسی رویوں یا جنسی عادات سے ہے۔ اُن سوالات کا جواب دیتے ہوئے آپ کو شائد اچھا نہ لگے۔

۲۔ آپ کا فون نمبر اور نام ہم اپنے ریکارڈ کے لئے لکھ رہے ہیں یہ کسی سے بھی شیئر نہیں کیا جائے گا۔

۳۔ ڈیٹا انالسیس میں آپ کی ذاتی تفصیل نہیں آئے گی۔

۴۔ اس ابت کا معمولی امکان ہے کہ آپ کا رزلٹ غلط آج جائے جو کہ کسی بھی ایچ آئی وی کی ٹیسٹ کٹ سے ہوسکتا ہے۔ اس سیلف ٹیسٹ کٹ کو WHO کے کوالٹی اشورنس پراسس سے گزرنے کے بعد استعمال میں لایا جا رہا ہے۔

۵۔ اگر آپ کا ایچ آئی وی کا رزلٹ مثبت یا پازیٹیو آگیا تو اُس سے آپ کو وقتی طور پر کافی ذہنی پریشانی ہوسکتی ہے۔ مگر ہم آپ کی اس پراسس میں پوری مدد کرینگے اور آپ کو نہ صرف ایچ آئی وی کی دوا دلوائیں گے بلکہ کاؤنسلنگ کا بھی انتظام کروائیں گے۔ ٹریٹمنٹ سینٹر پر کاؤنسلر موجود ہوتے ہیں۔

اس پروجیٹ کے رزلٹ کیسے استعمال ہونگے

اس پروجیٹ کے رزلٹ میں آپ یا کسی اور کی ذاتی تفصیل شامل نہں کی جائے گی اس پروجیکٹ کے رزلٹ سائنسی رسالوں میں اور سائنٹفک رپورٹس میں شیئر کیئے جائیں گے۔

کوئی معاوضہ

آپ کو 500 روپے کا موبائل کریڈیٹ ملے گا۔

رابطہ

PDF created with pdfFactory Pro trial version www.pdffactory.com
