# Supplementary material for: Acceptability and feasibility of HIV self-testing among transgender people in Larkana, Pakistan: Results from a pilot project
Source: PLoS One. 2022 Jul 8;17(7):e0270857. doi: 10.1371/journal.pone.0270857 (PMC9269381; doi:10.1371/journal.pone.0270857)
Supplement: S1 File — (ZIP) [file Ethical clearance letter.pdf › Supporting files/Ethical clearance letter.pdf]

BRIDGE CONSULTANTS FOUNDATION

COMMITTED TO PROMOTE PUBLIC HEALTH RESEARCH

BRIDGING SOCIAL, ECONOMIC AND HEALTH CONCERNS

Certified by :
Pakistan Centre for Philanthropy

26th October 2020

Dr. Arshad Altaf
Principal Investigator
HIVST Demonstration Study-Pakistan
WHO

Subject: Ethical clearance for "demonstration study to determine acceptable distribution model for HIV self-testing kits among key population in Pakistan"

Dear Dr. Arshad Altaf,

Thank you for submitting your research proposal for ethical review. Your proposal was reviewed in the committee's meeting held on 26th October 2020. The committee felt that self-testing can potentially lead to early diagnosis of HIV infection and the HIV positive person from key population group in Pakistan can be linked to treatment and further care which can help in reducing the ongoing transmission. However, we would like to emphasize on the following:

- Proper monitoring by site supervisors, as mentioned in the protocol.
- The outreach workers should follow all steps mentioned in the research protocol and the process must be monitored throughout the life of the project.
- Every effort should be made by outreach workers to transport and accompany all those found reactive on self-testing for confirmatory test and assist the HIV positive persons linked to treatment.
- Post-test counselling to all HIV negative persons should also be ensured.
- Confidentiality must be maintained by implementing CBOs and the investigators.

The project is granted ethical clearance for one year from today's date. Good luck to the implementing team.

Sincerely,

Muhammad Umer Abro
Chairperson
Ethical Review Committee

BRIDGE CONSULTANTS FOUNDATION

250/D, Block-6, PECHS, Nursery, Karachi-75400, Pakistan.
Phone : 021-34536151, 34532559 Fax : 92-21-34382758
E-mail : bridgeresearch_k@yahoo.com Website : http://www.bridgeresearch.org.pk
