# Supplementary material for: Acceptability and feasibility of HIV self-testing among transgender people in Larkana, Pakistan: Results from a pilot project
Source: PLoS One. 2022 Jul 8;17(7):e0270857. doi: 10.1371/journal.pone.0270857 (PMC9269381; doi:10.1371/journal.pone.0270857)
Supplement: Attachment — Submitted filename: PlosONE_REVIEWER COMMENTS_BN_.edited.pdf [file pone.0270857.s003.pdf]

Reviewer: Bernard Njau
Title: Acceptability and feasibility of HIV self-testing among transgender people in Larkana, Pakistan: results from a demonstration project

Manuscript Number: PONE-D-22-01177

Abstract:

(a) Under the Methods, I suggest the authors should use the word " trained transgender peer outreach workers " See line 9, page 2.
(b) The authors should add the Standard deviation after reporting the average age of participants was 25.5 years. See line 15 on page 2.
(c) The authors should provide numbers before reporting the percentages. For example, Just over one third, n/N (34.7%) had no formal education while 10.6% had attended at least five years of schooling. Over one third (38.6%) of participants were first-time testers. This should be corrected throughout the manuscript, including the result section. See lines 15-25 on page 2.
(d) The authors should report who provided the requested demonstration of the test procedure before completing HIVST. See line 21 on page 2.
(e) The authors should review the following statement "The demonstration required explaining the whole process a few minutes before the user used the kit or explaining and setting the kit and leaving the room" to make a clear meaning to the reader. See lines 21-23 on page 2.
(f) The authors should report How long did it take for linkage to care after receiving reactive HIVST results among the four participants? Out of the 4 participants how many were from unsupervised vs supervised HIVST?. See line23 on page 2.
(g) Conclusion: The authors should give a conclusion related to first-time testers results. See lines 27-29 on page 2.

Introduction:

(a). I suggest that the authors should describe the two types of HIVST strategies available (i.e., supervised vs. unsupervised HIVST). It should be added before the statement: " The World Health Organization (WHO)…". See line 23 on page 3.

(b) The authors should review the following statement " Globally, 94 countries have supportive HIVST policies are half of them (48) are now routinely implementing HIVST", to make it meaningful. See lines 24-25 on page 3.
(c) In the last paragraph the authors should justify why they selected the TGs and not PWIDs who have a higher HIV Prevalence? What are the expected benefits of doing this study and who will be the primary, secondary, and tertiary beneficiaries?. See lines 1-2 on page 4.

Methods:

(a) The authors should define what is a demonstration project in this study?. See line 2 on page 5.
(b) The authors should report if this study was a cross-sectional descriptive study using mixed methods? See line 1 on page 5.
(c) Since the study used a convenience sample, the authors should explain in the study limitation section, which potential biases could affect the study results. See line 3 on page 5.

Study settings

(a) I suggest the authors should provide more geographical, socio-economic, and number of health facilities/or HIV testing points and ownership information for readers to understand well the study settings? See lines 7-9 on page 5.

Study procedures

(a) I suggest the authors should provide information on who trained peers? Did they use any standard operating procedures/or manuals? How many were trained? Did they have any previous experience in HIV/AIDs related studies? The age range of the trained peers? See lines 19-23 on page 5.

(b) The authors should explain what methods did they use to demonstrate the use of HIVST kits? See lines 24-25 on page 5.

(c) Authors should give examples of the steps to take after receiving results. Also, they attach instructions materials used for training as supporting material. See line 24 on page 5.

(d) I suggest authors should briefly describe what the potential participants were informed about the study. See line 27 on page 5.

(e) The authors should report in the result section report: How many were approached and how many refused and how many-showed interest. See line 1 on page 6.

(f) I suggest that authors should give examples of HIV prevention services offered to participants. See line 20 on page 5, and line 11 page 6.

(g) The authors should be very specific to describe the benefits of the treatment. Was it ART, or?. See line 2 on page 6.

(h) I suggest that the authors should mention the source of the video and how long was the video? See line 9 on page 6.

(i) The authors should describe how did they define acceptability in this study? How did the authors define and measure feasibility in this study? See line 24 on page 6.

(j) Could the authors provide examples of the questions to assess: 1) acceptability, 2) ease of use of HIVST process and reading/interpreting their results; and 3) potential approaches and models for HIVST distribution among TGs and other key population groups. See lines 24-26 on page 6.

(k) One of the key concerns related to HIVST is social harm, including the possibility of committing suicide after receiving a reactive test result. Did the authors assess for suicidal ideation in this study and if not they should mention it as a study limitation.

Ethical considerations

(a) Authors should provide the Identification number of the ethical clearance certificate and the name of the relevant Ethical Review Committee. See line 28 on page 6.

Data management.

(a) There is very limited information here. The authors should provide a comprehensive description. The subheading should be data management and analysis. Then the authors should describe: 1) Quantitative data management and analysis, and 2) Qualitative data management and analysis. See lines 3-5 on page 7.

(b) The authors should explain in detail how did they conduct the content analysis technique, they should also justify why they used the content analysis technique instead of other qualitative data analysis techniques. See line 5 on page 7.

Results

(a) The authors should make changes as suggested in the abstract section above. See lines 2-18 on page 8.

(b) I suggest the authors should mention the initial concerns of participants as stated in the following statement ”participants had expressed initial concerns….” mentioned in line 14 on page 8.

(c) The authors should provide reasons why many participants preferred oral fluid kits instead of a finger prick. What barriers are associated with a finger prick? See line 17 on page 8.

Discussion:

(d) I suggest authors re-consider the use of the word” implementation research”. Does this study fit into the definition of implementation research? The authors should justify supporting the use of the word. See line 2 on page 9.

(e) I suggest that authors should provide existing evidence from other parts of the world supportive or contrary to their study findings.

(f) The authors should provide a citation at the end of the statement “…..a roadmap was developed” mentioned in line 9 on page 9.

(g) I suggest the authors should describe why the implementation has lagged as mentioned in line 10 on page 9.

(h) The authors should provide a citation at the end of the statement “ HIV tests performed in 2020 were 3,552,819 compared to 8,844,382 in 2019. . See lines 12 to 13 on page 9.

Study limitations:

(i) The authors should provide additional limitations related to using convenient sampling. See line 1 on page 10.

(j) I suggest authors should provide a reason to support the following statement "However, we expect similar findings in urban settings with greater awareness and availability of services" mentioned in lines 2-3 on page 10.

Summary:

(k) The authors should be very specific on areas that National programmes and implementers can use the information for effective programming. See lines 9-10 on page 10.

Table 1:

a) Usually table 1 is for socio-demographic characteristics…Authors can have another table 2 for 1) acceptability of HIVST; 2) ease of use of HIVST process and reading/interpreting their results; and 3) potential approaches and models for HIVST distribution among TGs and other key population groups.

b) Authors should be specific about “other details”.
