# Supplementary material for: Acceptability and feasibility of HIV self-testing among transgender people in Larkana, Pakistan: Results from a pilot project
Source: PLoS One. 2022 Jul 8;17(7):e0270857. doi: 10.1371/journal.pone.0270857 (PMC9269381; doi:10.1371/journal.pone.0270857)
Supplement: Attachment — Submitted filename: Responses to reviewer 1_Arshad Altaf.docx [file pone.0270857.s004.docx]

**Responses to reviewer 1**

| **Reviewer comment** | **Author response** | **Text change** |
| --- | --- | --- |
| **Reviewer 1** |  |  |
| I think this is an interesting and important paper sharing the considerations on HIVST in Pakistan among transgender populations. This paper could be framed in a way that acknowledges the HIVST knowledge gaps in EMRO and among transgender people more so. Throughout the term demonstration project is used - and it is unclear how the authors define this and differentiated it from a pilot study or implementation science research. It is unclear how the national programme was involved and will be involved moving forward, though there are statements about this study informing policy work and providing evidence to support implementation. This needs to be supported more fully in the paper. | Thank you for your valuable comment.  We have revised the title to a “pilot project” as it seems a more appropriate term for this study.  The national programme was fully on board during the project’s inception, field implementation and manuscript development. | Acceptability and feasibility of HIV self-testing among transgender people in Larkana, Pakistan: results from a pilot project  Although HIVST has been included in the national policy in Pakistan since 2018 to date neither implementation has taken place, nor have any products registered. HIVST response in Pakistan is largely externally donor funded. In 2018, the national programme and other partners organized a national consultation on HIVST which supported the introduction of HIVST and led to the development of a roadmap in this direction. However, subsequently, there has been no progress to date given the lack of resources. The national programme and other partners are now working collectively to introduce HIVST in 2022. |
| **Abstract** | | |
| I would clarify from the beginning you are focusing on transgender people and be clear this is because of their risk and being key populations. | We agree and the text has been clarified to address this | Owing to the high risk of HIV among the transgender (TGs) persons they are among the highest risk group for HIV in Pakistan, yet knowledge of HIV status remains low in this key population group. We conducted a pilot project to assess the acceptability and feasibility of distributing HIVST to TGs in Larkana |
| I don't know if you need the brand name (OraQuick) in the abstract - just say oral fluid. | Thanks for this suggestion. We have removed the brand name | “One oral fluid HIVST kit per person was distributed free of cost in the community by trained transgender peer outreach workers (ORW). Demographic characteristics of participants were collected. The ORW followed up with phone calls to record if HIVST kits were used, the results and whether any assistance was required.” |
| Clarify if distribution was one kit per person. | This information has been included |  |
| You note a (as in 1) follow-up call placed indicate that this was indeed just one and improve clarity on language as results indicates there was other follow-up time points and mechanisms. | Thanks for this suggestion. This has been clarified within the limitation of word count. Detailed information is in the Methods section |  |
| Felt like you could have noted the estimated PLHIV aware of status and TG testing coverage. Could make your results read more compelling on the potential benefits given the substantial testing and treatment gaps in Pakistan. | Point well taken. Because of the limited words allowance, we cannot add this in the background section. |  |
| **Introduction** | | |
| Unclear what you mean by Pakistan's data showing 84% increase in infections. Need reference and clarity. | Thank you. We have addressed it along with the reference. | There has been an 84% increase in the number of new HIV infections in Pakistan between 2010 and 2020. Despite the increase, less than a quarter (22%) of all people living with HIV (PLHIV) were aware of their HIV status in 2020 and only 12% of PLHIV were on antiretroviral treatment (ART). |
| I would mention KP briefly and directly focus on TG from the beginning in the intro - you can mention applicability for other KP in discussion. Data on other groups not as informative and confusing if trying to prioritize TG as a unique contribution of this paper. Globally there hasn't been as much data on HIVST use among TG - and this is important to capture insights and preferences for this group in addition to translating to broader understanding on HIVST in Pakistan. How are TG reached or not reach currently that should be explained - so the gaps and potential comes out more in addition to the general programme needs overall. | Thank you for pointing this out. We have revised it as per your suggestion. | According to UNAIDS estimates, HIV prevalence among TGs in 2020 was estimated to be 5.5%. Awareness of and access to HIV services including HIV testing remain low among TG. According to national behavioral surveillance, in 2016-2017, only a third (34%) of all TGs reported having ever tested for HIV and less than half (43%) know where to get tested for HIV. |
| Clarify what you mean by new now - HIVST has been recommended since 2016 so it isn't new globally so adjust that point on "new approaches". | Thank you for highlighting it. We have revised the sentence. | The World Health Organization (WHO) has recommended HIVST as an HIV testing approach first in 2016 and an updated recommendation in 2019 |
| Reasons why the product hasn't been introduced could be addressed more and how this study address gaps. Could be addressed in aims and objectives. With the country aiming to implement in 2022 - how this informs anything in addition to WHO guidance needs to be clearer. It seems like the country should have been able to implement the guidelines without research on acceptability - should be more the how to implement. So some background on why this study was needed should be clearer. | Relevant sentences from the discussion section moved up in the introduction section. | Although HIVST has been included in the national policy in Pakistan since 2018, to date, neither implementation has taken place, nor have any products registered. HIVST response in Pakistan is primarily externally donor funded. In 2018, the national programme and other partners organized a national consultation on HIVST, which supported the introduction of HIVST and led to the developing of a roadmap in this direction. However, there has been no progress to date, given the lack of resources. The national programme and other partners are now working collectively to introduce HIVST in 2022. |
| I don't think you need to say "Global Fund grant" and should acknowledge national programme leadership. | Noted with thanks.  The primary author has worked in the NACP as well as with the NACP in developing two National AIDS Strategies and has provided technical assistance multiple other times. Unfortunately, the national programme has shown poor leadership in implementing an effective HIV response in the country. However, we do not want to say this in the manuscript. We hope that the esteemed reviewer will understand.  **THIS IS FROM GLOBAL AIDS MONITORING 2020 REPORT:**  “The HIV response in Pakistan continues to be challenged by certain “external” challenges that include weak coordination between stakeholders, inadequate inter-provincial information sharing, collation reporting and utilization mechanisms, variations in HIV interventions and lack of effective community engagement. The HIV programs in the country are pre-dominantly donor-dependent (mainly the Global Fund grants) with limited key-population specific and geographic coverage. Challenges like access to HIV services, stigma and discrimination, sexual and gender violence, human rights abuses and lack of community and social support in addition to capacities of the CBOs to execute community-based prevention programmes." |  |
| The history of the outbreak and testing in Larkana may be useful to note more - as there were many challenges with testing reported during this time. | Thank you. We have added the information. | In 2019, Larkana was the center of an HIV outbreak that primarily affected children. Children (ages 5 to 16 years) accounted for 86% of all HIV infections, with unsafe injection practices being a major source of transmission. |
| **Methods** | | |
| - The sentence should read "A convenience sample of 150 TGs was recruited using oral fluid HIVST kits. OraQuick HIV Self-Test (OraSure Technologies Inc., Pennsylvania, US) was used in this study." | Thank you. We have revised the sentence. | A convenience sample of 150 TGs was recruited using oral fluid HIVST kits. OraQuick HIV Self-Test (OraSure Technologies Inc., Pennsylvania, US) was used in this study. The kits were donated free of cost by the manufacturer for this study. |
| The limited resources should be addressed separately and may be better placed in limitations and discussion for justifying why you used the methods you did. | Thank you. We have adjusted as per your suggestion. |  |
| - Make this sentence into two sentences "The inclusion criteria were adults aged 18 years or above, self-identifying as TG and residing in Larkana. All enrolled participants reported that they were HIV negative or had not been tested in the past six months." | Thank you for the suggestion. We have revised this sentence. | The inclusion criteria were adults aged 18 years or above, self-identifying as TG and residing in Larkana. All enrolled participants reported that they were HIV negative or had not been tested in the past six months. |
| - Points on consent should be in the ethics section for clarity on how participants were consented into the study and with ethical approval section explained. | Thank you for pointing this out.  We would like to draw the attention of the esteemed reviewer that it is mentioned in the “ethical considerations” section that all participants provided written informed consent. It is also mentioned in the “inclusion and exclusion criteria” section that those participants who were unable to read instructions were excluded. |  |
| - Was consent written or verbal? Clarify how you ascertained the information about their ability to read instructions. | Thank you for pointing this out.  It is already mentioned that written informed consent was obtained. We have also added the words “in the Urdu language” further clarifying that those who were unable to read the Urdu language were excluded. | Participants were excluded if they were HIV positive or on ART, unable to provide consent, unable to read instructions in the Urdu language, or reported prior HIVST use. |
| - Acknowledging some role and support of national programme in addition to CBO would be valuable – as they should have some role or role in future that needs to be noted.. | Thank you for pointing this out. We have added a sentence in the methods. | This study was designed as a pilot in consultation with the National AIDS Control Programme (NACP), so the lessons could inform the future more comprehensive programmatic implementation of HIVST in this population. |
| - Further clarify if participants always provided just 1 kit. Efforts to document if anyone who took a kit home gave it to someone else. | Thank you for pointing this out. This was not done and has been added as a limitation. |  |
| - What happened if the phone call led to no answer - was that the last attempt of contact? clarify. | Thank you for raising this point. The relevant sentence has been added. | If the first phone call led to no answer, another call was made the same day. |
| - The way data was captured doesn't come out clearly and leaves questions in results - was this a questionnaire that people filled in - were asked - some semi-structured questions? Greater clarity needed. | Thank you for raising this point. We have added sentences which now clarify this ambiguity. | Each participant was administered a two part questionnaire. The first part was completed at the time of enrolment and included quantitative questions related to demographics, risk behavior, and prior testing history. The second part which was completed after participants performed HIVST included three open ended questions related to HIVST acceptability (overall experience and ease of use; perceived ability to perform HIVST) and preferences for accessing HIVST in the future. The feasibility was defined as the successful implementation of the intervention with use of peer workers. The open ended questions were:   1. How was your experience of participating in this project? 2. Do you think MSM and TGs can do HIVST on their own in Pakistan? What difficulties do you foresee and how can those be fixed? 3. In your opinion, how should HIVST kits be distributed among key population in Pakistan.   Those completing HIVST at home were asked these questions during follow up (see later). |
| **Results** | | |
| - clarify oral fluid HIVST preference - this seems to be compared to professional fingerstick. So that should be revised in results and clarified if this is result or more of authors discussion point. | Thank you for pointing this out.  We have added the word “oral” in the paragraph where responses to follow up questions are mentioned. We hope this will suffice. | In response to open ended follow up questions related to HIVST use and acceptability, 139 (92.6%) reported oral HIVST to be an acceptable HIV testing approach. Seventeen (11.3%) participants had expressed initial concerns and anxiety about the use of self-testing but found the overall HIVST process to be satisfactory. |
| **Discussion** | | |
| - This is one of few HIVST studies in TG. Suggest highlighting hat - and the importance of this. - Registration and access to products should be raised as a key point for moving forward with the results - as well as the national programme aims and ability to move this CBO model forward. | Thank you for pointing this out.  Relevant sentences were added in the opening and second paras of the discussion. | This is the first HIVST research in Pakistan where participants performed HIVST. This is also one of the few HIVST studies conducted among TGs. Peer-based HIVST distribution was found to be feasible and acceptable among TGs in a non-urban setting. The majority of participants could perform HIVST with instructions provided without the need for additional assistance. Many participants were first time testers, and four new HIV infections were diagnosed and linked to ART. |
| - Address the context of Larkana and community testing challenges in the recent outbreaks and how HIVST applicability specifically here when there were issues documented on efforts to scale-up testing in the district using existing models. | Thank you for highlighting this. | After the 2019 HIV outbreak, a mass testing campaign was initiated where a number of factors related to HIV testing were overlooked ranging from lack of pre and posttest counseling and ethical considerations related to privacy and confidentiality (also from personal communication with AAS, field manager of an NGO based community engagement project in Ratodero). The availability of HIVST in such a scenario would have helped in alleviating some of those concerns. |
| - Linkage to ART in Pakistan is going to be a broader issue - i wouldnt say that the findings alleviate concern but confirm what has been found in other studies that people who test positive want to link. But access to ART and easier ways for KP to link remain a gap and a priority in Pakistan. Strategies to overcome this challenge in scaling up ART access and uptake need to continue to be prioritized. How does HIVST fit into this and other references to work in the country for addressing this? | Thank you for your comment. We have deleted the sentence related to “alleviate concerns” and added new sentences as per the suggestion of the reviewer. | These findings are consistent with evidence from other settings showing peer navigation can improve linkage to care with HIVST compared to HIVST alone. Where feasible such approaches can be considered to improve linkage. Nevertheless, delayed initiation of ART and challenges related to access to treatment and care by PLHIV and key population have been documented in Pakistan. It is only prudent to timely address these issues in order to increase the number of HIV positive persons receiving treatment and care. HIVST can also help in increasing the testing rates, particularly among key population who are residing in rural settings such as Larkana and its adjoining areas. |
| - We have seen literacy and education be a difference in urban vs rural, that could be worth noting that it is likely urban areas would have better feasibility. However some preferences for facility-based and blood testing have been observed in urban settings. Given limited access to testing in less urban areas, HIVST interest could be higher in Larkana. | Thank you for raising this point. We agree with this useful comment.  A sentence has been added to address this useful comment. | HIVST can also help in increasing the testing rates, particularly among the key population who are residing in rural settings such as Larkana and its adjoining areas. |
| - Limitation on the incentive / compensation and relying on self-report means that they might not have been reporting accurately and self-report has a bias. |  | We provided free HIVST kits and a small incentive to participants as well as to peer workers. The element of self-reporting bias also exists. We are not sure if results will be similar in real world setting in the absence of incentives or if key populations have to pay for HIVST kits. To maximize the uptake and benefit of HIVST kits to the key population it is pertinent that these kits are affordable, preferably free of cost. |
| The concluding statements should focus on relevance for TG and KP, engaging with national programme, but it isnt clear that this evidence was "much needed" and will change things in Pakistan. Can that be addressed? Anything on what this work has helped inform and progress that it is true an 150 person study is leading to some policy changes and action should be added, otherwise it seems like it remains unclear why Pakistan hasnt implemented WHO guidelines that have been in place since 2016 and it's 2022. | Thank you for pointing this out.  We added TGs in the concluding statement. Also, in our preparatory call which was organized by the WHO Country Office in December 2020 with NACP and UNAIDS Pakistan, the need for a demonstration project was specifically pointed out. | In summary, this demonstration project has provided much-needed local and context specific evidence which supports HIVST availability to key populations and its acceptability among TGs. National programmes and implementers can use the information for effective programming especially the peer or community based approach for HIVST distribution. HIVST availability should be prioritized for key populations in Pakistan. Further implementation can generate lessons for other populations and relevant delivery models. |
